# Transposable elements as novel therapeutic targets for PARPi-induced synthetic lethality in PcG-mutated blood cancer^∗^

**DOI:** 10.1182/blood.2025028560

**Published:** 2025-07-08

**Authors:** Bernd B. Zeisig, Chiou-Tsun Tsai, Clemence Virely, Tsz Kan Fung, Ali Tuğrul Akin, Estelle Troadec, Bo Jiao, I. Richard Thompson, Priscilla Nga Ieng Lau, Nanjun Li, Andriani Charalambous, Larissa Bomfim, Jennifer Lynch, Athina Georgiou, Robbert Hoogeboom, Claire Lynn, Si-Yi Zhang, Piers E. M. Patten, Cynthia L. Fisher, Anna Schuh, Seishi Ogawa, Ghulam Mufti, Mohammad M. Karimi, Chi Wai Eric So

**Affiliations:** 1 Comprehensive Cancer Centre, King’s College London, London, United Kingdom; 2 Department of Haematological Medicine, King’s College Hospital, London, United Kingdom; 3 Department of Medical Biology, Faculty of Medicine, Istinye University, Istanbul, Turkey; 4 State Key Laboratory for Medical Genomics, Shanghai Institute of Hematology, National Research Center for Translational Medicine at Shanghai, Ruijin Hospital affiliated to Shanghai Jiao Tong University School of Medicine, Shanghai, China; 5 School of Biomedical Engineering, The University of British Columbia, Vancouver, Canada; 6 Department of Oncology, University of Oxford, Oxford, United Kingdom; 7 Department of Pathology and Tumor Biology, Graduate School of Medicine, Kyoto University, Kyoto, Japan

## Abstract

Loss-of-function (LoF) mutations frequently found in human cancers are generally intractable by classical small molecule inhibitor approaches. Among them are mutations affecting Polycomb-group (PcG) epigenetic regulators, enhancer of zeste homolog 2 (EZH2) and Additional sex combs like 1 (ASXL1), frequently found in hematological malignancies of myeloid or lymphoid lineage, and their concurrent mutations associates with particularly poor prognosis. Although there is a clear need to develop novel and effective treatments for these patients, the lack of appropriate disease models and mechanistic insights have significantly hindered the progress. Here, we show that genetic inactivation of Asxl1 and Ezh2 in murine hematopoietic stem/progenitor cells results in highly penetrant hematological malignancies as observed in corresponding human diseases. These PcG proteins regulate both coding and noncoding genomes, leading to marked reactivation of transposable elements (TEs) and DNA damage responses in PcG LoF–mutated cells, which create a novel vulnerability for poly(ADP-ribose) polymerase (PARP) inhibitor (PARPi)–induced synthetic lethality. Using both mouse models and primary patient samples, we demonstrate that Asxl1/Ezh2-mutated cells are highly sensitive to PARPis that induce excessive DNA damage and significantly extend disease latency. Intriguingly, the observed PARPi sensitivity can be specifically overridden by reverse transcriptase inhibitors that interrupt target site–primed reverse transcription and life cycle of TEs. This mechanism is contrastingly different from the current concept of BRCAness associated PARPi-induced synthetic lethality, which largely rely on deficient homologous recombination, and is independent on reverse transcriptase inhibitors. Together, this study reveals a novel application and mechanism of PARPi-induced synthetic lethal targeting of blood cancers with reactivated TEs such as those carrying PcG epigenetic mutations.

## Key Points

• Epigenetic reactivation of TEs creates a novel and tractable vulnerability in PcG-mutated leukemia.
• Novel PARPi-induced synthetic lethality via target site–primed reverse transcription associated with reactivated TEs.

## Visual Abstract

**Figure:**
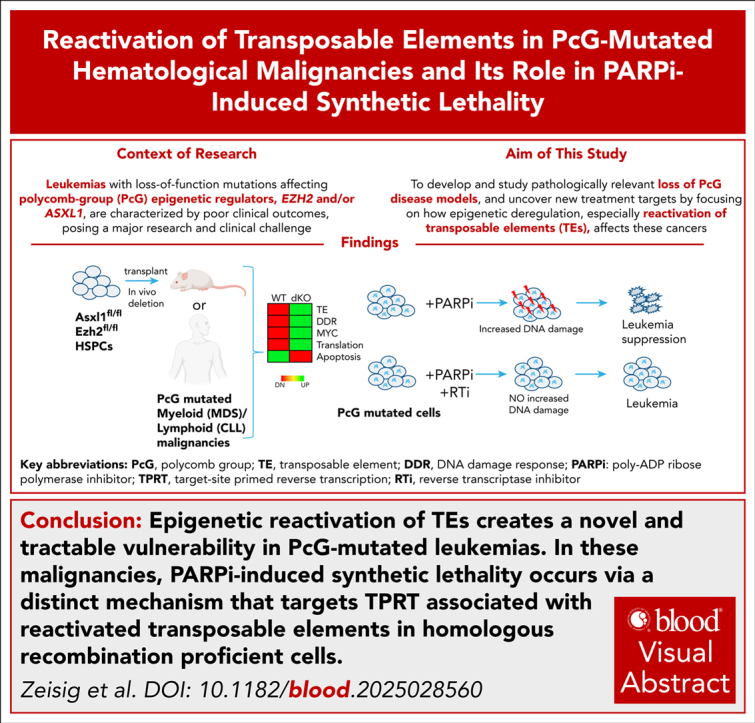

## Teaser

Leukemias harboring loss-of-function mutations in Polycomb-group epigenetic regulators, such as EZH2 and ASXL1, are associated with poor clinical outcomes and remain a major therapeutic challenge. Zeisig et al demonstrate that these mutations drive leukemogenesis by reactivating transposable elements, areas of DNA sequence that can move from one locus to another within the genome, leading to DNA damage and creating a vulnerability to poly(ADP-ribose) polymerase (PARP) inhibitors. Unlike traditional PARP inhibitor sensitivity linked to defective DNA repair, this new mechanism depends on transposable element activity and can be abrogated with reverse transcriptase inhibitors, offering a promising therapeutic strategy for these hard-to-treat leukemias.

## Introduction

Loss-of-function (LoF) mutations in Polycomb repressor complexes (PRCs) affecting enhancer of zeste homolog 2 (EZH2) and Additional sex combs like 1 (ASXL1) are frequently identified in both myeloid and lymphoid neoplasms, and correlate with poor prognosis.^1^, ^2^, ^3^, ^4^, ^5^, ^6^ *ASXL1* exon 12 mutation resulting in frameshift and nonsense transcripts is one of the most common mutations accounting for up to 45% of patients with myelodysplastic syndrome (MDS)/myeloproliferative neoplasm (MPN) and chronic myelomonocytic leukemia (CMML).^1^^,^^7^, ^8^, ^9^ Furthermore, ASXL1 LoF mutation has recently been reported as one of the most significant poor prognostic markers in patients with chronic lymphocytic leukemia (CLL).^10^^,^^11^ Interestingly, forced expression of a truncated ASXL1 protein predicted from the frameshift mutation could also promote the development of myeloid malignancies in a transgenic mouse model, suggesting a potential pathological function of the putative truncated protein, although such proteins remain to be detected in patients.^12^, ^13^, ^14^, ^15^ Suppression of EZH2 activity has been proposed as a key oncogenic function associated with ASXL1 mutation,^15^^,^^16^ which is consistent with the findings of EZH2 LoF mutation in ∼10% of patients with MDS/MPN and CMML.^17^ Paradoxically, in contrast to the classical mutual exclusivity of mutations with direct overlapping functions, EZH2 LoF mutation frequently coexists with ASXL1 mutation (cBioportal: MDS, adjusted *P* < .001; MPN, adjusted *P* = .032) in human patients with MDS/MPN^1^^,^^17^ and CMML^18^ associated with a worse clinical outlook, urging the need for thorough experimental investigations to resolve this dilemma.^17^^,^^19^^,^^20^ While it has been a major challenge to target LoF mutations by small molecule inhibitor approaches when mutated proteins often cannot be found and/or do not have a clear role in driving the disease, the lack of appropriate disease models has further compounded the issue and significantly impeded the progress in studying the underlying mechanisms of these diseases and development of novel effective therapeutics.

Transposable elements (TEs) that account for almost half of the human genome,^19^^,^^21^, ^22^, ^23^ but are historically regarded as ancient junk sequences,^24^^,^^25^ have been reported in recent years to be reactivated in driving disease development,^26^ and multiple cellular processes including gene expression,^27^ DNA damage, and immune responses.^28^, ^29^, ^30^, ^31^, ^32^, ^33^ To restrict and regulate their activities, TEs are normally suppressed by multiple epigenetic mechanisms including DNA methylation, histone modifications, and noncoding RNAs.^25^ Although histone 3 lysine 9 (H3K9) methylation has been viewed as a major regulator for TE activity, recent literature also suggest a key regulatory function of H3K27 methylation by PRCs on TEs.^22^^,^^23^ Consistently, TE expression can be altered as a result of epigenetic drug treatments in cancer.^26^^,^^34^^,^^35^ Strikingly, TE reactivation in cancer cells can potentially drive anticancer immunity,^28^, ^29^, ^30^, ^31^, ^32^, ^33^ and generates novel immunogenic antigens,^34^ although relevant preclinical and clinical therapeutic data are needed to assess their full potentials. On the other hand, reactivated TEs can also induce replicative stress and excessive DNA damage, which we hypothesize that may create a specific vulnerability and can potentially be exploited by synthetic lethality approaches.^36^

The prevalence of LoF mutations affecting multiple Polycomb group (PcG) proteins in hematological malignancies provides an excellent opportunity to study and test our hypothesis. To this end, the current study modeled the loss of PcG mutations in hematopoietic stem/progenitor cells (HSPCs) that resulted in various hematological malignancies like those observed in humans. Importantly, we also demonstrate that specific epigenetic reactivation of TEs in these cells offers a novel therapeutic avenue for poly(ADP-ribose) polymerase inhibitor (PARPi)–induced synthetic lethality via suppression of PARP-mediated protection of single-strand DNA (ssDNA) break during target site–primed reverse transcription (TPRT), which can be abolished by reverse transcriptase inhibitors (RTis).

## Methods

### Human samples

Frozen viable human samples were obtained from the UK CLL biobank and the King's College London (KCL) biobank with approval from the local ethics committee. All tissue samples would have gotten informed consent before banking and use.

### Mice

Compound *Asx1*^fl/f*l*^*Ezh2*^fl/fl^ Rosa26-CreER mice were generated by backcrossing *Ezh2*^tm1Tara^ (MGI2661097)^37^ Gt(ROSA)26Sor^*tm1(cre/ESR1)Tyj*^ (MGI: 3699244) with *Asxl1*^tm1c(EUCOMM)Wtsi^^38^ in C57BL/6J (CD45.2) background, in which critical exons (exon 16-19 in *Ezh2* and exon 3 in *Asxl1*) are flanked with loxP sites. All mice were held and kept in specific pathogen-free biological services unit at King’s College London, and experiments were performed according to the protocols in the project license (P8B15B1E1) under the Animals (Scientific Procedures) Act 1986.

### Flow cytometry and antibodies

Cell staining was performed as previously described^39^ (supplemental Table 1, available on the Blood website).

### Comet assay

The comet assay kit (catalog no. 4250-050-K; R&D Systems, Minneapolis, MN) was used according to the manufacturer instructions. When assessing the degree of DNA damage, at least 15 randomly selected microscopic fields were imaged per slide covering a minimum of 500 cells in total.

### γH2AX staining

Staining was performed as previously described.^40^

### Next-generation sequencing and bioinformatic analysis

RNA sequencing (RNA-seq) and Cleavage Under Targets and Release Using Nuclease (CUT&RUN) were performed as previously described^41^ (supplemental Table 1). Next-generation sequencing (NGS) PE150 RNA-seq reads were aligned using the STAR aligner.^42^ TE locations were identified using Repeatmasker^43^ and used by the featureCount of Rsubread^44^ to give read counts at all known TEs. Differential expression was carried out using DESeq2.^45^ CUT&RUN analysis was conducted using the CUT&RUNTools pipeline^46^ with default parameters.

Detailed information is provided in supplemental Methods.

## Results

### Loss of Asxl1 and Ezh2 leads to aberrant expansion of HSPCs and development of MDS/MPN and B-lymphoproliferative disease in vivo

**Figure 1.**
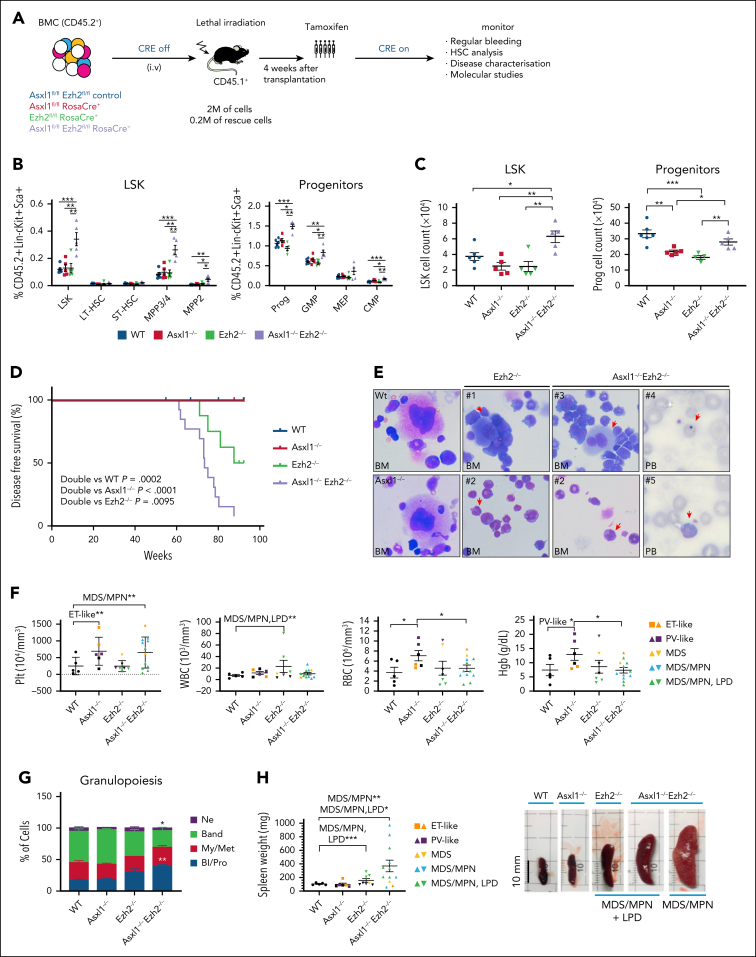
**dKO of Asxl1 and Ezh2 leads to the development of myelodysplastic disorders with high penetrance.** (A) Schematic diagram of the primary transplantation experiment. (B) Percentages of LSK, myeloid progenitors (Lin^–^c-kit^+^Sca1^–^), and their subpopulations: LT-HSCs (CD48^–^CD150^+^), ST-HSCs (CD48^–^CD150^–^), MPP3/4 (CD48^+^CD150^–^), MPP2 (CD48^+^CD150^+^), GMP (CD16/32^+^CD34^+^), CMP (CD16/32^–^CD34^+^), and MEP (CD16/32^–^CD34^–^) in BM from indicated mutant mice 4 weeks after tamoxifen treatment are shown. WT, n = 6; all other groups, n = 5. (C) Absolute cell counts of CD45.2^+^ LSK and myeloid progenitors in BM are shown. WT, n = 6; all other groups, n = 5. (D) Kaplan-Meier survival curves of WT (n = 8), *Asxl1*^–/–^ (n = 6), *Ezh2*^–/–^ (n = 8), and *Asxl1*^–/–^*Ezh2*^–/–^ (n = 13) mice in primary transplantation. (E) Representative images of May-Grunwald Giemsa staining of BM from indicated mice and PB smears from *Asxl1*^–/–^*Ezh2*^–/–^ mice. Myeloid dysplastic cells such as monolobated megalokaryocytes (1), pseudo–Pelger-Huët anomalies (2), binucleated megakaryocytes (3), Howell Jolly bodies (4), and giant platelets (5) were found in the mice with myelodysplasia. (F) Blood counts of WT, Asxl1^–/–^, Ezh2^–/–^, and Asxl1^–/–^Ezh2^–/–^ mice with indicated disease phenotypes in primary transplantation. Statistical analysis is shown for the indicated groups, unless otherwise stated. (G) The percentages of hematopoietic cells Bl/Pro, My/Met, band cells, and Ne in granulopoiesis from WT and KO mice with MDS/MPN phenotypes. WT and Asxl1^–/–^, n = 3 each; EZH2^–/–^, n = 2; Asxl1^–/–^Ezh2^–/–^, n = 5. (H) Spleen weights (left) and representative photos of spleens (right) for the indicated genotypes and disease phenotypes. WT, n = 5; Asxl1^–/–^, n = 6; Ezh2^–/–^, n = 8; Asxl1^–/–^Ezh2^–/–^, n = 12. Plots show mean ± standard error of the mean (SEM). ∗*P* < .05; ∗∗*P* < .01; ∗∗∗*P* < .001, unpaired *t* test. Bl/Pro, blasts/promyelocytes; CMP, common myeloid progenitor; ET, essential thrombocythemia; GMP, granulocyte-monocyte progenitor; Hgb, hemoglobin; Lin^–^, lineage negative; LT-HSC, long-term HSC; MEP, megakaryocyte–erythroid progenitor; MPP3/4, multipotent progenitor 3/4; My/Met, myelocytes/metamyelocytes; Ne, neutrophils; Plt, platelets; PV, polycythemia vera; RBC, red blood cells; ST-HSC, short-term HSC; WBC, white blood cells.

To study the impacts of loss of Asxl1 and Ezh2 on hematopoiesis, CD45.2^+^ bone marrow (BM) cells from single or compound *Asxl1*^*fl/fl*^*Ezh2*^*fl/fl*^ mice harboring an inducible Rosa26-CreER allele were transplanted together with CD45.1^+^ control rescue cells into lethally irradiated CD45.1^+^ recipient syngeneic mice for assessment of their roles in normal and malignant hematopoiesis (Figure 1A). Although we observed myeloid-biased repopulation in the Ezh2 and Asxl1/Ezh2 knockout (KO) groups (supplemental Figure 1A-C), analysis of HSPCs uncovered that concurrent depletion of *Asxl1* and *Ezh2* enhanced the number of Lineage^−^c-kit^+^Sca1^+^ (LSK) cells, in particular multipotent progenitor 2 to multipotent progenitor 4 populations and myeloid progenitors (Lineage^−^c-kit^+^Sca1^−^) in BM (Figure 1B-C). Moreover Asxl1^−/−^Ezh2^−/−^ LSK cells displayed accelerated cell cycle progression upon Ki67 staining (supplemental Figure 1D), partially accounting for the observed expansion of HSPCs.

Although the wild-type (WT) control animals did not develop any noticeable hematological malignancies, almost all *Asxl1*/*Ezh2* double KO (dKO) mice succumbed to fatal MDS/MPN, associated with marked splenomegaly and extramedullary hematopoiesis in the presence of B-cell lymphoproliferative disorders (LPDs) in some animals (Figure 1D-H; supplemental Figure 1E-G; supplemental Table 2). This contrasts with the single KO mice in which *Asxl1* KO mice were largely normal with mild polycythemia vera–like or essential thrombocythemia–like hematopoietic phenotypes noted in some animals, whereas *Ezh2* KO resulted in low penetrant MDS/MPN LPDs with extended disease latency (Figure 1D-H, supplemental Figure 1E-G; supplemental Table 2). May-Grunwald Giemsa staining revealed markedly dysplastic features such as abnormal megakaryocytes (monolobated nuclei and bilobed megakaryocytes), giant platelets, Howell Jolly bodies, and pseudo–Pelger-Huët anomalies (hyposegmented neutrophils) in the BM and/or peripheral blood (PB) of *Ezh2* single KO and *Asxl1/Ezh2* dKO diseased mice, indicating trilineage dysplasia that was particularly noticeable in myeloid and megakaryocytic cell types (Figure 1E). Consistent with the respective disease phenotypes, *Asxl1* KO diseased mice had elevated red blood cells, hemoglobin, and/or platelet levels in line with polycythemia vera–like or essential thrombocythemia–like phenotypes (Figure 1F; supplemental Figure 1E), whereas *Asxl1/Ezh2* dKO showed left-shifted granulopoiesis with a significant increase in the percentage of myeloblasts and promyelocytes and a significant decrease of band cells (Figure 1G) with thrombocytosis (Figure 1F). Splenomegaly was clearly noted in mice that developed MDS/MPN with or without LPDs (Figure 1H). Together, these data indicate that inactivation of Asxl1 and Ezh2 in HSPCs results in development of aggressive myeloid malignancies as observed in human patients.

### Asxl1/Ezh2 dKO LPD leads to aggressive CLL

**Figure 2.**
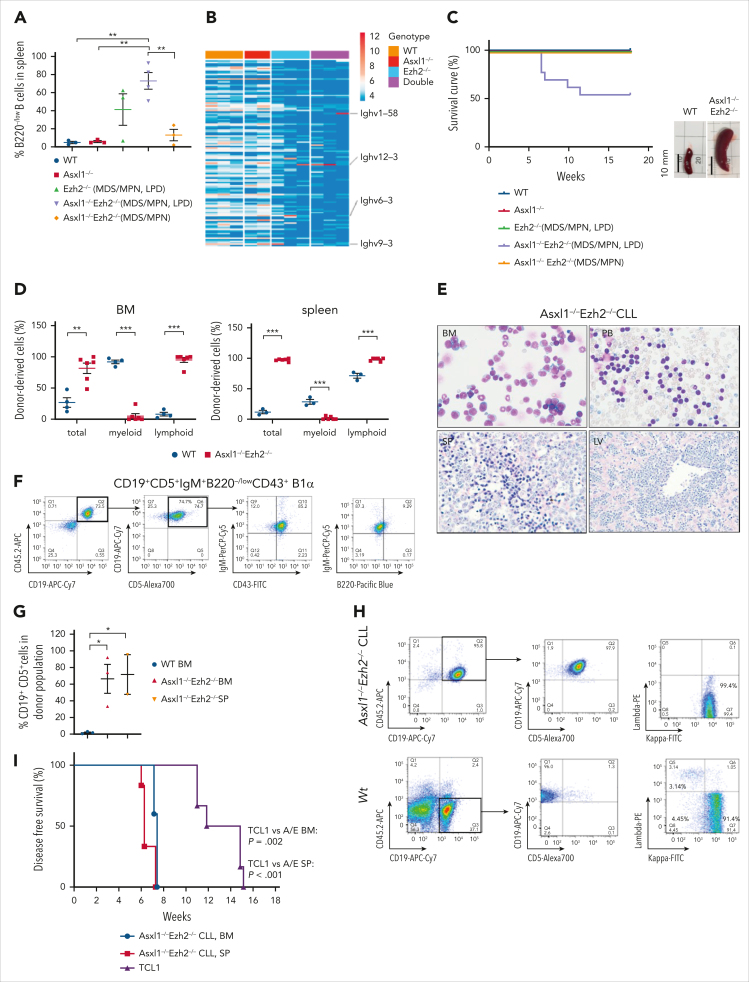
**Serial transplantation of Asxl1^–/–^Ezh2^–/–^ cells lead to aggressive CLL.** (A) The percentage of B220^–/low^ B cells in CD45.2^+^ splenocytes from mice that received primary transplant with indicated genotypes and disease phenotypes are shown. Asxl1^–/–^Ezh2^–/–^ (MDS/MPN, LPD), n = 4; all other sample types, n = 3. (B) The heat map shows normalized read counts of 124 IGHV in the BM from mice that received primary transplant with the indicated genotype. The monoclones with substantial expansion are pointed out. (C) Kaplan-Meier survival curves of secondary transplants; mice were transplanted with BM cells harvested from mice that received primary transplant with the indicated genotypes and disease phenotypes. WT, n = 5; Asxl1^–/–^, n = 5; Ezh2^–/–^ (MDS/MPN, LPD), n = 10; Asxl1^–/–^Ezh2^–/–^ (MDS/MPN, LPD), n = 13; Asxl1^–/–^Ezh2^–/–^ (MDS/MPN), n = 3 mice. Representative images of the SPs are shown. (D) Engraftments of total donor cells (CD45.2^+^) and the ratios of myeloid and lymphoid populations in the donor population from the BM and SP (as indicated) of WT (BM, n = 4; SP, n = 3) and *Asxl1*^–/–^*Ezh2*^–/–^ dKO secondary CLL (n = 6) mice. (E) May-Grunwald Giesma staining of BM (original magnification x40) cells and PB (x40) smears, and hematoxylin and eosin staining of the SP (x20) and LV (x10) of Asxl1^–/–^Ezh2^-/-^ dKO secondary CLL mice. (F) Representative flow cytometry profiling of CLL cells in BM from Asxl1^–/–^Ezh2^–/–^ dKO secondary CLL mice. (G) The dot plot represents the percentages of CD19^+^CD5^+^ cells in donor cells from BM (n = 3) and SPs (n = 2) from Asxl1^–/–^Ezh2^–/–^ dKO secondary CLL mice compared with WT BM (n = 3). (H) Proportions of κ/λ light chains in CD19^+^ donor splenocytes isolated from Asxl1^–/–^Ezh2^–/–^ dKO secondary CLL or WT mice as indicated. (I) Kaplan-Meier survival curves of tertiary transplants. Mice were transplanted with BM cells and splenocytes from a secondary *Asxl1*^–/–^*Ezh2*^–/–^ CLL mouse or splenocytes from a secondary TCL1 CLL mouse. Plots show mean ± SEM. ∗*P* < .05; ∗∗*P* < .01; ∗∗∗*P* < .001, unpaired *t* test. A/E, Asxl1^-/-^Ezh2^-/-^; LV, liver; SP, spleen; TCL1, T-cell leukemia/lymphoma 1A oncogene.

Further analysis of LPDs in the diseased mice revealed a major expansion of B220^−/low^ non-T(CD4^−^CD8^−^) lymphoid cells (Figure 2A; supplemental Figure 2A) that expressed (1) a specific gene signature matching those of B1α cells (supplemental Figure 2B), the putative origin of CLL^47^^,^^48^; and (2) clonal immunoglobulin heavy variable domains (Figure 2B), suggesting clonal CLL-like disease. Indeed, we identified clonal rearrangement of IGHV-IGHD-IGHJ rearrangements (Table 1; supplemental Figure 2C-F) with unmutated IGHV genes (≥98% identity to germ line counterpart) in mice with CLL-like disease, indicating a monoclonal LPDs with unmutated IGHV genes. Intriguingly, 3 of 6 LPD mice shared an obvious stereotyped subset with highly homologous IGHV-IGHD-IGHJ gene rearrangement, displaying significant similarity on the variable domain heavy chain CDR3, a feature found in human patients with CLL,^49^ suggesting a common antigen for the clonal expansion and pathological relevance of the PRC2-mutated CLL-like model, analogous to the corresponding human disease (Table 1).

**Table 1.** **Clonality of LPDs**

Genotype	ID	Clone	VH	D	JH	Mutation	HCDR3
Ezh2 KO	1092-17	Mono	V6-3	D3-1	JH3	(U)	T G G G S W F A Y
Ezh2 KO	1092-24	Mono	V1-52	D2-8	JH4	(M)	T R G G Y S G H Y G A V D Y
Ezh2 KO	1092-20	Mono	V12-3	D4-1	JH1	(U)	A G D R T G Y W Y F D V
A/E dKO	1093-7	Mono	V12-3	D1-1	JH1	(U)	A R D S Y G Y W Y F D V
A/E dKO	1078-26	Mono	V12-3	D4-1	JH1	(U)	A G D I G G Y W Y F D V
A/E dKO	1092-26	Mono	V1-58	D2-5	JH4	(U)	A R S D Y S N Y V S Y Y Y A M D Y

Analysis of IGHVDJ from splenocytes of MDS/MPN LPD mice.

A/E dKO, Asxl1/Ezh2 dKO; D, diversity region; HCDR3, heavy chain complementary determining region; ID, identifier; JH, joining region heavy chain; M, mutated IGHV; Mono, monoclone; U, unmuted IGHV; VH, variable domain heavy chain.

To further assess their leukemic property, serial transplantation assay was performed using BM cells from primary hosts with WT, single KO, or dKO cells. Although no MDS/MPN-like disease phenotype was observed in mice that received secondary transplantation, aggressive CLL-like diseases developed with short latency in approximately half of the animals (6/13) transplanted with *Asxl1*^*−/−*^*Ezh2*^*−/−*^ LPD cells but not *Ezh2*^*−/−*^ LPD cells (Figure 2C). The CLL mice with significant expansion of donor-derived B cells exhibited severe organomegaly (splenomegaly and hepatomegaly), high BM cellularity, leukocytosis, and anemia referring to a high-risk stage III according to Rai staging system^50^ (Figure 2D; supplemental Figure 2G). Morphologically typical CLL cells with clumpy nuclei and a rim of visible cytoplasm were identified in the CLL mice (Figure 2E). Consistently, flow cytometry revealed the CLL with CD19^+^B220^−/low^CD5^+^IgM^+^CD43^+^ B1α cell phenotype (Figure 2F-G). Moreover, all CD19^+^ B cells were κ light chain restricted, indicating a clonal expansion of CLL cells (Figure 2H), which had the identical IGHV-IGHD-IGHJ gene rearrangement as the primary CLL cells (supplemental Figure 2H).

To further characterize the *Asxl1*^*−/−*^*Ezh2*^*−/−*^ CLL model, we compared it with the commonly used Eμ-TCL1 transgenic CLL mouse model using BM cells for tertiary BM transplantation. Compared with mice with TCL1 cells that developed CLL at ∼12 weeks with moderate splenomegaly and a high level of CD19/CD5 double-positive cells in the PB, mice receiving either BM or splenocytes from *Asxl1*^*−/−*^*Ezh2*^*−/−*^ CLL mice succumbed to disease in 6 weeks with organomegaly in the spleen, liver, and lymph nodes, and moderate level of circulating CD19/CD5 double-positive cells (Figure 2I; supplemental Figure 2I-K). In addition to lymphocytosis, anemia, and thrombocytopenia were also identified in *Asxl1*^*−/−*^*Ezh2*^*−/−*^ CLL mice (supplemental Figure 2L). Together with the organomegaly, the aggressive phenotype of *Asxl1*^*−/−*^*Ezh2*^*−/−*^ CLL could be defined as high-risk Rai stage IV in tertiary transplantation, whereas the tertiary transplanted TCL1 CLL phenotype was like that of patients with intermediate Rai stage II, without anemia and thrombocytopenia. Consistent with a more aggressive form of CLL,^51^^,^^52^ cleaved CLL cells were only identified in *Asxl1*^*−/−*^*Ezh2*^*−/−*^ CLL mice (supplemental Figure 2M). Together, these data depict a novel murine CLL model driven by loss of Asxl1 and Ezh2.

### Asxl1/Ezh2 dKO cells are characterized by activation of TE expression and DNA repair pathways

**Figure 3.**
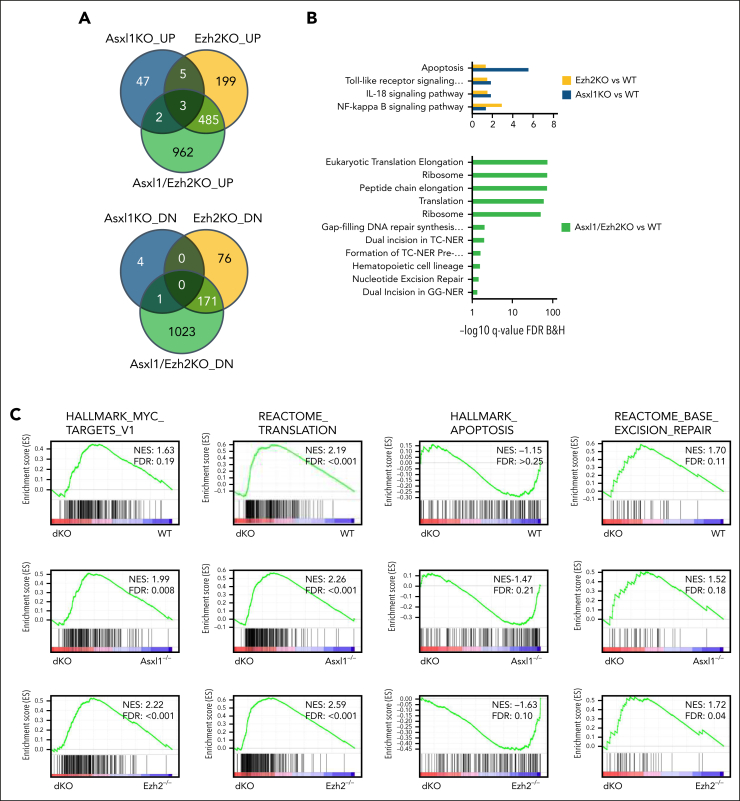

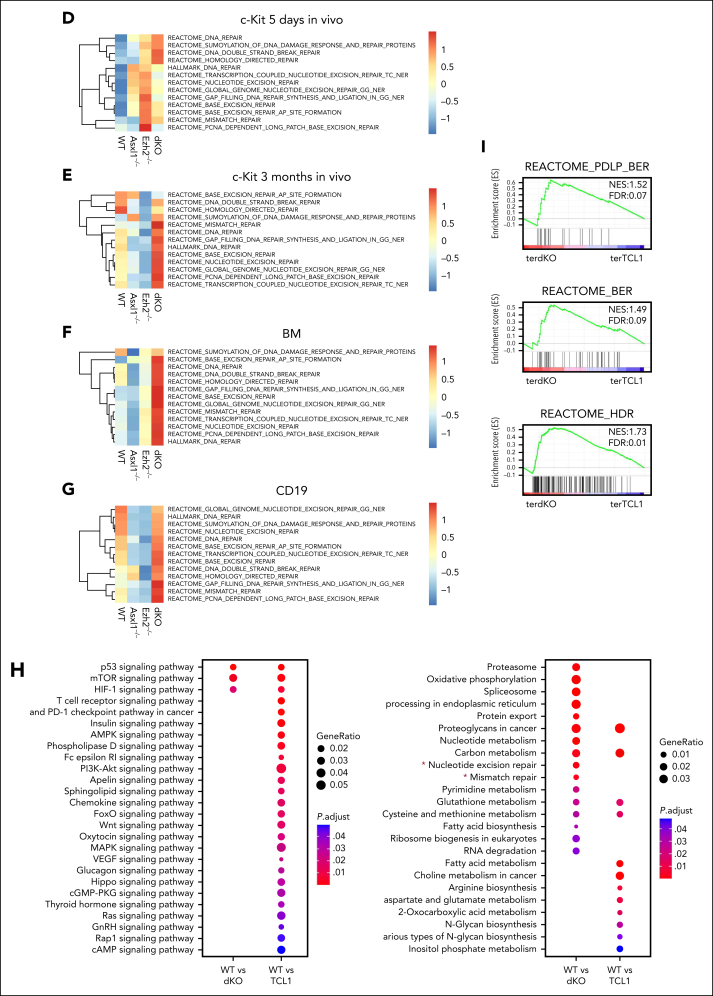

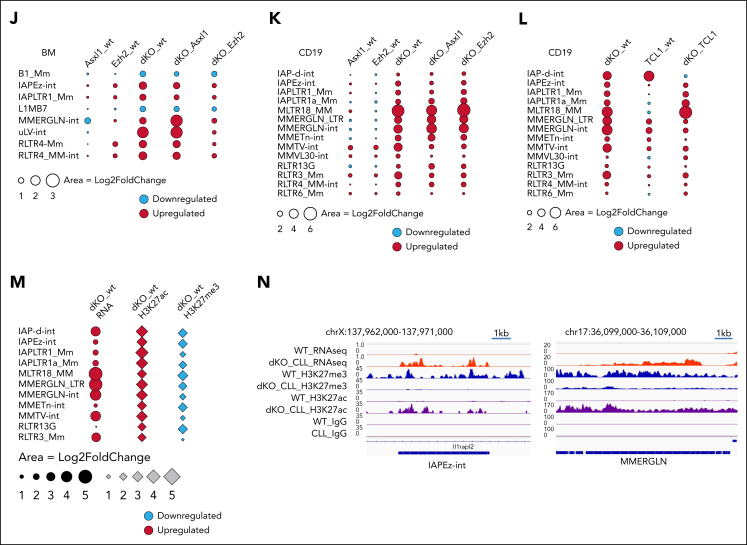
**DNA repair pathways are uniquely active in ERV re-expressing Asxl1^–/–^Ezh2^–/–^ dKO cells.** (A) Venn diagram showing the overlaps of differentially expressed genes (adjusted *P* < .05) identified in the data set derived from the BM of mice that received primary transplant with the indicated genotype, by comparing each genotype, *Asxl1*^–/–^, *Ezh2*^–/–^ MDS/MPN LPD, and *Asxl1*^–/–^*Ezh2*^–/–^ dKO MDS/MPN LPD mice with WT. (B) The bar charts show example pathways identified in gene ontology analysis comparing Asxl1^–/–^ vs WT or Ezh2^–/–^ vs WT (top) and Asxl1^–/–^Ezh2^–/–^ dKO vs WT (bottom). (C) Example GSEA plots for the indicated pathways in the indicated comparisons are shown. (D-G) Gene set variant analysis (GSVA) for the indicated samples was performed and mean scores for Reactome and Hallmark DNA repair gene sets for each genotype are plotted in the heat map. Different RNA-seq data sets were used for the analysis, c-kit^+^ 5 days after start of in vivo deletion (D), c-kit^+^ 3 months after in vivo deletion (E), BM (F), and CD19^+^ splenocytes (G). (H) Dot plots show selected results for Kyoto Encyclopedia of Genes and Genomes pathway analysis using genes expressed in CLL in the indicated comparisons (WT vs tertiary Asxl1^–/–^Ezh2^–/–^ dKO CLL or WT vs tertiary TCL1 CLL). DNA repair pathways are marked with a star. (I) GSEA results for selected DNA repair gene sets for tertiary Asxl1^–/–^Ezh2^–/–^ dKO CLL vs tertiary TCL1 CLL are shown. (J) Dot plot showing the differential expression of the indicated TE families in BM RNA-seq data from mice that received primary transplant, comparing KO vs WT and dKO vs single KO as indicated. (K) Similar dot plot as shown in panel J but using CD19^+^ splenocyte RNA-seq data from mice that received primary transplant. (L) Dot plot showing the differential expression of the indicated TE families using CD19^+^ splenocytes isolated from tertiary Asxl1^–/–^Ezh2^–/–^ dKO CLL, tertiary TCL1 CLL mice, or WT mice as control. (M) The plot shows the integration of differential expression analysis using CD19^+^ RNA-seq data and quantitative H3K27me3 and H3K27ac CUT&RUN data for the indicated TE families in CD19^+^ splenocytes isolated from tertiary Asxl1^–/–^Ezh2^–/–^ dKO CLL mice or WT mice as control. (N) The genome tracks show examples of differentially expressed individual ERVs (RNA-seq top 2 tracks) and the H3K27me3 and H3K27ac levels in WT and tertiary Asxl1^–/–^Ezh2^–/–^ dKO CLL mice as indicated. The last 2 tracks show control IgG enrichment. H3K27ac, H3K27 acetylation; H3K27me3, H3K27 trimethylation; IgG, immunoglobulin G.

We performed RNA-seq analysis with BM (Figure 3A-C) or CD19^+^ splenocytes (supplemental Figure 3A-C) harvested from all 4 groups of mice that received primary transplant. Gene ontology and gene set enrichment analysis (GSEA) revealed that specific activation of apoptosis pathway found in single Asxl1^*−/−*^ and single Ezh2^*−/−*^ cells was absent in *Asxl1*^*−/−*^*Ezh2*^*−/−*^ cells, which instead prominently expressed transcriptional programs with well-established roles in oncogenic transformation such as Myc and translation (Figure 3B-C; supplemental Figure 3B-C). Specific activation of these oncogenic pathways and suppression of apoptosis in Asxl1/Ezh2 dKO cell may explain the synergism between these 2 mutations. Interestingly, we also observed activation of multiple DNA damage repair (DDR) pathways in these cells, indicating excessive DNA damage, an established feature for reactivation of TEs^28^ (Figure 3B-C; supplemental Figure 3B-C).

Gene set variant analysis was used as a complementary method to further assess their activities in BM CD19^+^ and c-kit^+^ HSPCs harvested from the BM 5 days or 3 months after tamoxifen treatment. As a result, Asxl1^*−/−*^Ezh2^*−/−*^ cells consistently showed the highest expression of multiple DNA repair pathways in most of the comparisons except at the 5-day time point when transient Cre-recombination–mediated DDR was noted in all the floxed groups (Figure 3D-G). To further assess the specificity of the observed enrichment in DDR pathways, we compared RNA-seq from CD19^+^ spleen cells isolated from Asxl1/Ezh2 dKO or TCL1 CLL mice with WT CD19^+^ spleen cells. Although both CLL models shared several overlapping pathways (supplemental Figure 3D), DNA repair pathways were only found in Asxl1/Ezh2 dKO CLL (Figure 3H), which was further validated in GSEA analysis (Figure 3I), consistently suggesting excessive DNA damage being induced in Asxl1/Ezh2 dKO cells. Because DDR dysregulation can be potential therapeutic target for cancer,^53^ we focused our analyses on the underlying mechanisms for deregulated DDR.

To this end, we closely examined candidate pathways previously reported being regulated by the PcG proteins including p53-p21–, coactivator-associated arginine methyltransferase 1-, and AKT serine/threonine kinase 1 mammalian target of rapamycin–dependent pathways, however, none of them was significantly deregulated in all relevant comparisons (supplemental Figure 3E, data not shown). Then we turned our attention to TEs with known capability of inducing DNA damage. We first mapped the RNA-seq data from BM cells (Figure 3J) or CD19^+^ splenocytes (Figure 3K) harvested from primary mice against all TEs in the genome to determine the differentially expressed families of TEs (adjusted *P* ≤ .01; log_2_ fold change of ±1.5 or higher; maximum reads per million of ≥1000). Although a moderate upregulation on TE subfamilies were noted in single KO (Figure 3J), their expressions were significantly higher in Asxl1/Ezh2 dKO cells (Figure 3J-K; supplemental Figure 3F), suggesting that combined inactivation of both PcG genes leads to a strong reactivation of TEs. Whereas no DNA TEs was found to be upregulated, key family members of all 3 endogenous retrovirus (ERV) classes, including intracisternal A-type particles, MMERGLN, murine leukemia virus, and long-terminal repeat retrotransposon families were activated in Asxl1/Ezh2 dKO across different comparisons. We note that TE derepression was more significant in Ezh2 single mutant than those in Asxl1 single-mutant BM cells (Figure 3J-K). Importantly, the level of TE derepression correlated well with DDR, in which Ezh2 single-mutant BM cells showed stronger DDR activity than Asxl1 single mutant (Figure 3F), indicating a bigger contribution by EZH2 to TE depression and DDR in BM cells. However, the deregulation of TE expression by PcG is likely cell type specific because we observed a largely similar impact upon inactivation of individual PcG protein in CD19^+^ spleen cells (Figure 3K). Further analysis of the RNA-seq data from mice that received tertiary transplantation and developed advanced CLL confirmed that ERV expression was indeed specifically upregulated in Asxl1/Ezh2 dKO as compared with the control WT CD19^+^ B cells or TCL1 CD19^+^ CLL cells (Figure 3L). Mechanistically, CUT&RUN sequencing on CD19^+^ splenocytes revealed a decrease in H3K27 trimethylation repressive mark and an increase in the H3K27 acetylation activation mark for many ERV subfamilies differentially expressed in Asxl1/Ezh2 dKO (Figure 3M-N; supplemental Figure 3G), resulting in their transcriptional derepression. The epigenetic landscape of Asxl1/Ezh2 dKO CLL at TE elements is also different as compared with TCL1 CLL (supplemental Figure 3H), which had higher H3K27 trimethylation and lower H3K27 acetylation marks. These results consistently suggest epigenetic deregulation underlying the observed transcriptional derepression in Asxl1/Ezh2 dKO, which could be detected in the 3-month time point (supplemental Figure 3I-J), before notable disease onset. TPRT is a well-characterized mechanism for attempted reintegration of TEs into host genomes, which will produce ssDNA intermediates that are protected by PARP and replication protein A complex.^54^ Given that PARP activity is required to safeguard transiently exposed ssDNA during TPRT, we hypothesize that suppression of PARP may induce excessive DNA damage in these cells and represent an effective novel treatment for the diseases.

### Asxl1/Ezh2 dKO cells are highly sensitive to PARP inhibition

**Figure 4.**
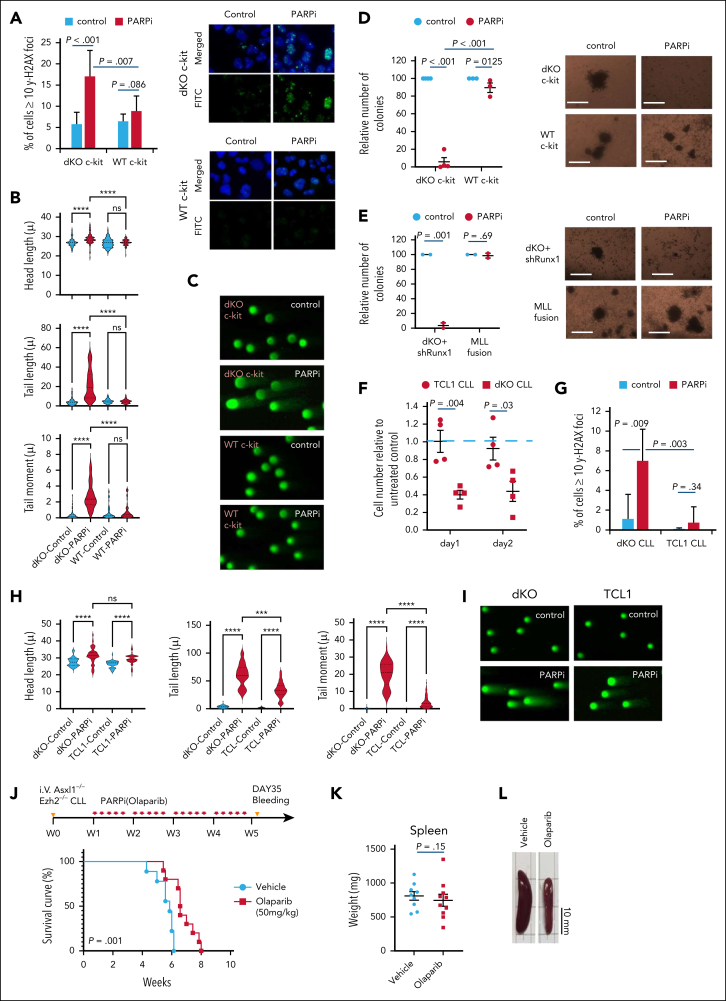
**Mouse Asxl1^–/–^Ezh2^–/–^ dKO cells are highly sensitive to PARPi.** (A) The bar chart (left) shows the percentage of cells with ≥10 γH2AX foci for the indicated cell type with or without PARPi treatment. Representative images (right) of γH2AX foci (green) and their merge with DAPI (4′,6-diamidino-2-phenylindole; blue) are shown. (B) Comet assay was performed after treatment with or without PARPi on in vitro–deleted Asxl^–/–^Ezh2^–/–^ dKO c-kit^+^ or WT c-kit^+^ cells and head length, tail length, and tail moment are shown in the violin plots. (C) Example comet pictures for control and PARPi-treated cells are shown. (D-E) The relative number of colonies for in vitro–deleted Asxl1^–/–^Ezh2^–/–^ c-kit^+^ cells (n = 4) and WT c-kit^+^ cells (n = 3) (D) and Asxl1^–/–^Ezh2^–/–^ Runx1KD MDS (n = 2) and MLL-transformed cells (n = 2) (E) with or without PARPi treatment are shown (left). Representative colony pictures are shown (right). (F) The dot plot shows the cell numbers relative to untreated control (blue dotted line) for Asxl1^–/–^Ezh2^–/–^ dKO CLL (n = 4) and TCL1 CLL (n = 4) after 1 or 2 days of PARPi treatment. (G) The bar chart shows the percentage of cells with ≥10 γH2AX foci upon in vitro treatment with or without PARPi. (H) Comet assay was performed after treatment with or without PARPi on Asxl^–/–^Ezh2^–/–^ dKO CLL or TCL1 CLL cells and head length, tail length, and tail moment are shown in the violin plots. (I) Example comet pictures for control and PARPi-treated cells are shown. (J) Schematic diagram of the experimental plan to treat tertiary Asxl1^–/–^Ezh2^–/–^ dKO CLL cells in vivo with the indicated drugs (vehicle, 10%; HBC, n = 9; olaparib, 50 mg/kg; olaparib, n = 10) (top). Kaplan-Meier survival curves of Asxl1^–/–^Ezh2^–/–^ dKO CLL mice treated with vehicle or olaparib treatment indicated (bottom). (K) Weight of the spleens of mice at the end of experiment for the indicated groups. Vehicle, n = 9; Olaparib, n = 10. (L) Representative photos of the spleens from Asxl1^–/–^Ezh2^–/–^ dKO CLL mice treated with indicated drugs. Plots show mean ± SEM, ∗∗∗*P* < .001, ∗∗∗∗*P* < .001, unpaired *t* test. FDR, false discovery rate; FITC, fluorescein isothiocyanate; NES, normalized enrichment score; ns, not significant.

In order to test this hypothesis, we first assessed the nature of DNA breaks in dKO cells by neutral and alkaline comet assays. Consistent with our hypothesis, we observed that although similar level of double-strand DNA (dsDNA) breaks found in both Asxl1/Ezh2 dKO and WT control, Asxl1/Ezh2 dKO cells however carried a significantly higher level of ssDNA breaks during TPRT, which are protected by PARP from progressing into devastating dsDNA breaks (supplemental Figure 4A). Then we investigated whether Asxl1^*−/−*^Ezh2^−/−^ c-kit^+^ HSPCs (Figure 4A-D) were indeed sensitive to PARPi (olaparib) treatment using in vitro culture and clonogenic assay as previously described.^40^ Although a concentration of up to 1μM olaparib exhibited minimal effects on normal HSPCs (supplemental Figure 4B), Asxl1^−/−^Ezh2^−/−^ cells were highly sensitive to PARPi-induced DNA damage as evidenced by detection of excessive DNA damage gH2Ax foci (Figure 4A) and dsDNA breaks as showed in comet assays (Figure 4B-C; supplemental Figure 4C). Importantly, PARPi resulted in ∼90% reduction in their colony numbers (Figure 4D) but had little or no detectable effect on WT or control PARPi-resistant MLL-fusion transformed cells^40^ (Figure 4A-D; supplemental Figure 4C-D). Similar results were obtained using a different PARPi, veliparib that also exhibited specific suppression on Asxl1^−/−^Ezh2^−/−^ but not WT HSPCs (supplemental Figure 4E). Next, we tested the PARPi vulnerability of Asxl1^−/−^Ezh2^−/−^ cells using another new Asxl1^−/−^Ezh2^−/−^ Runx1KD mouse model that mimic RUNX1 haplodeficiency frequently coexisting with the PcG LoF mutations in patients with MDS.^1^ Knocking down the expression of Runx1 in Asxl1^−/−^Ezh2^−/−^ c-kit^+^ cells produced an even more aggressive MDS/MPN disease with a shorter latency than Asxl1^−/−^Ezh2^−/−^ control (supplemental Figure 4F-H). Strikingly, PARPi treatment very significantly suppressed the colony-formation ability in Asxl1^−/−^Ezh2^−/−^Runx1KD MDS cells and again had no notable impact on control MLL-fusion transformed cells (Figure 4E), suggesting a specific PARPi vulnerability was created and remained in Asxl1^−/−^Ezh2^−/−^ cells despite the presence of additional genetic events.

In addition to myeloid leukemia, we also tested the PARPi sensitivity of CLL cells (Figure 4F-L). Although olaparib treatment did not exert any notable effect on TCL1 CLL cells, it significantly induced apoptosis and reduced the number of Asxl1^−/−^Ezh2^−/−^ CLL cells (Figure 4F; supplemental Figure 4I). Similar results were observed using 2 independent PARPis, veliparib and talazoparib (supplemental Figure 4J). Consistently, significant enrichments of γH2AX DNA damage foci (Figure 4G) and dsDNA breaks (Figure 4H-I) were specifically observed in Asxl1^−/−^Ezh2^−/−^ cells as compared with untreated controls and TCL1 CLL cells upon PARPi treatment (Figure 4H-I; supplemental Figure 4K). To further elucidate the in vivo efficacy of PARPi treatment, mice transplanted with Asxl1^−/−^Ezh2^−/−^ CLL were treated with olaparib 1 week after tertiary transplantation (Figure 4J). Blood analyses at day 35 (end of treatment) revealed that although red blood cell, hemoglobin, and platelet counts in olaparib treatment groups were similar to untreated controls, the CLL counts in PARPi-treated animals were significantly reduced (supplemental Figure 4L). Furthermore, in week 4, mice treated with vehicle started developing severe organomegaly and circulation of CLL cells in the PB, whereas olaparib significantly delayed the disease onset (Figure 4J-L). Together, these results reveal a specific DNA damage vulnerability in Asxl1^−/−^Ezh2^−/−^ leukemia cells that can be targeted by PARPi.

### Derepression of TEs is required for PARPi sensitivity in Asxl1/Ezh2 dKO cells

**Figure 5.**
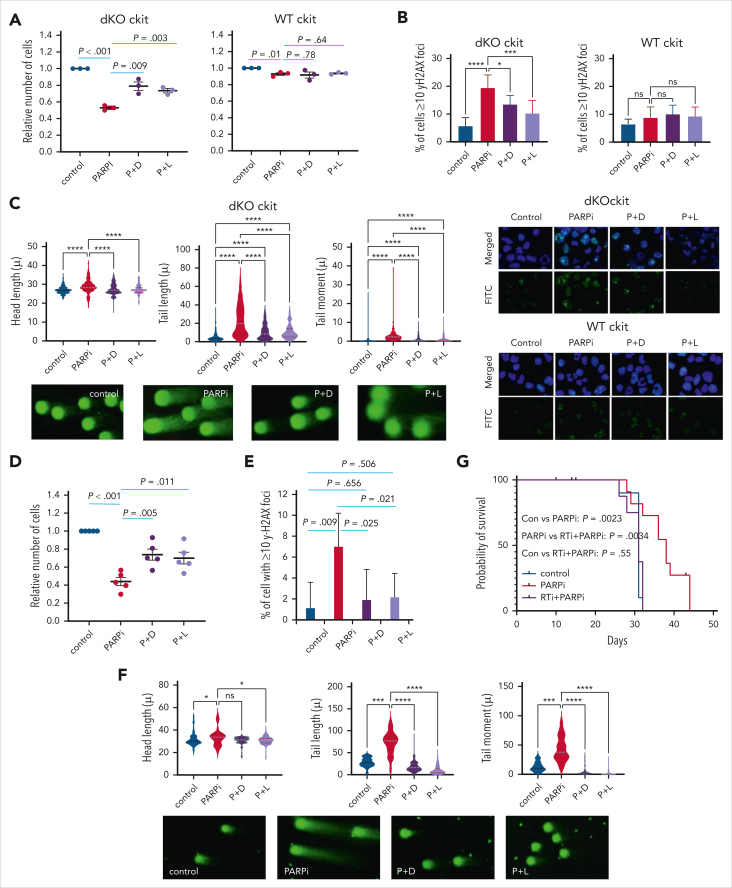
**ERV reactivation drives PARPi sensitivity in murine Asxl1^-–/–^Ezh2^–/–^ dKO cells.** (A) The relative number of in vitro–deleted Asxl^–/–^Ezh2^–/–^ dKO c-kit^+^ (n = 3) or WT c-kit^+^ cells (n = 3) are shown following the indicated in vitro treatment (P+D and P+L). (B) The bar chart shows the percentage of cells with ≥10 γH2AX foci upon the indicated treatment for Asxl^–/–^Ezh2^–/–^ dKO c-kit^+^ or WT c-kit^+^ cells (top). Representative images of γH2AX foci (green) and their merge with DAPI (blue) are shown (bottom). (C) Comet assay was performed after the indicated treatments of Asxl^–/–^Ezh2^–/–^ dKO c-kit^+^ cells and head length, tail length, and tail moment are shown in the violin plots (top). Example comet pictures are shown (bottom). (D) The relative number of Asxl1^–/–^Ezh2^–/–^ dKO CLL cells (n = 5) are shown after the indicated treatment. (E) The bar chart shows the percentage of cells with ≥10 γH2AX foci after the indicated treatment of Asxl1^–/–^Ezh2^–/–^ dKO CLL cells. (F) Comet assay was performed after the indicated treatment on Asxl^–/–^Ezh2^–/–^ dKO CLL cells and head length, tail length, and tail moment are shown in the violin plots (top). Example pictures are shown (bottom). (G) Kaplan-Meier survival curves of mice transplanted with Asxl1^–/–^Ezh2^–/–^ dKO CLL and treated in vivo as indicated are shown. Control, n = 11; PARPi, n = 13; RTi + PARPi, n = 12. Plots show mean ± SEM, ∗*P* < .05, ∗∗∗*P* < .001, ∗∗∗∗*P* < .001, unpaired *t* test. ns, not significant; P+D, PARPi + didanosine; P+L, PARPi + lamivudine.

Mechanistically, PARPi sensitivity can be due to suppression of homologous recombination (HR) genes^55^^,^^56^ as we have previously reported for acute myeloid leukemia (AML) driven by repressive oncogenic transcription factors such as RUNX1/RUNX1T1 (AML1-ETO).^40^ However, in line with our reactome, GSEA and differential gene expression analyses in which we found that key HR genes including Brca1 and Rad51 were mostly unchanged or even upregulated in dKO cells (supplemental Figure 3E), functional studies assessing Rad51 repair foci (supplemental Figure 5A), parylation (supplemental Figure 5B), and HR functionality (supplemental Figure 5C) further demonstrated HR competency in Asxl1^−/−^Ezh2^−/−^ dKO cells, consistently indicating an alternative underlying mechanism, which we hypothesize is caused by reactivation of TEs. Reverse transcriptase is an essential enzyme specifically required for the replication life cycle of retroviral TEs and TPRT.^24^^,^^25^ To confirm the functional link between TE reactivation and PARPi sensitivity, we first sought to investigate whether inhibition of the key reverse transcription step by 2 independent nucleoside/nucleotide RTis, namely didanosine and lamivudine, would suppress the observed PARPi sensitivity. As expected, c-kit^+^–enriched HSPCs from Asxl1^−/−^Ezh2^−/−^ mice but not WT mice were highly sensitive to PARPi treatment (Figure 5A-C; supplemental Figure 5D-F). Strikingly, PARPi sensitivity was partially but significantly reversed by cotreatment with either dianosine or lamivudine, which otherwise had no impact on HSPCs derived from WT mice. Importantly, RTi treatment did not affect PARPi sensitivity of AML1-ETO–transformed cells (supplemental Figure 5G-I), whose PARPi sensitivity results from suppression of HR genes,^40^ consistently indicating that RTis do not generally counteract PARPi treatment but exert a specific function targeting TPRT in Asxl1^−/−^Ezh2^−/−^ dKO cells. Conversely, excessive γH2AX DNA damage foci and dsDNA breaks induced by PARPi in Asxl1^−/−^Ezh2^−/−^ cells were partially but significantly reversed by RTis, suggesting that reactivation of TE plays a key role in the observed PARPi sensitivity (Figure 5A-C). Importantly, Asxl1^−/−^Ezh2^−/−^ Runx1KD cells remain PARPi sensitive in a RTi-dependent manner (supplemental Figure 5J), suggesting that additional mutations do not diminish the key role of reactivated TEs in the observed PARPi sensitivity. Next, we performed similar treatments on Asxl1^−/−^Ezh2^−/−^ CLL cells (Figure 5D-G). As expected, PARPi treatment was effective in suppressing the CLL cells by inducing excessive DNA damage (Figure 5D-F; supplemental Figure 5K). Similarly, we observed marked rescue of PARPi killing in Asxl1^−/−^Ezh2^−/−^ CLL cells by different RTi, which resulted in reduced DNA damage and improved CLL cells survival upon PARPi treatment (Figure 5D-F). Finally, we also assessed in vivo combination treatment on mice transplanted with Asxl1^−/−^Ezh2^−/−^ CLL cells. As a result, although RTi alone did not affect the disease latency consistent with a limited role of TE reactivation in driving disease development, it dramatically reversed efficacy of PARPi treatment and shortened the survival time, which was virtually identical to the PARPi-untreated control group (Figure 5G; supplemental Figure 5L). Together, these results demonstrate a direct functional link between reactivation of TEs and PARPi sensitivity in Asxl1^−/−^Ezh2^−/−^ leukemia cells.

### Human leukemia with ASXL1/EZH2 mutations exhibits TE reactivation and PARPi sensitivity

**Figure 6.**
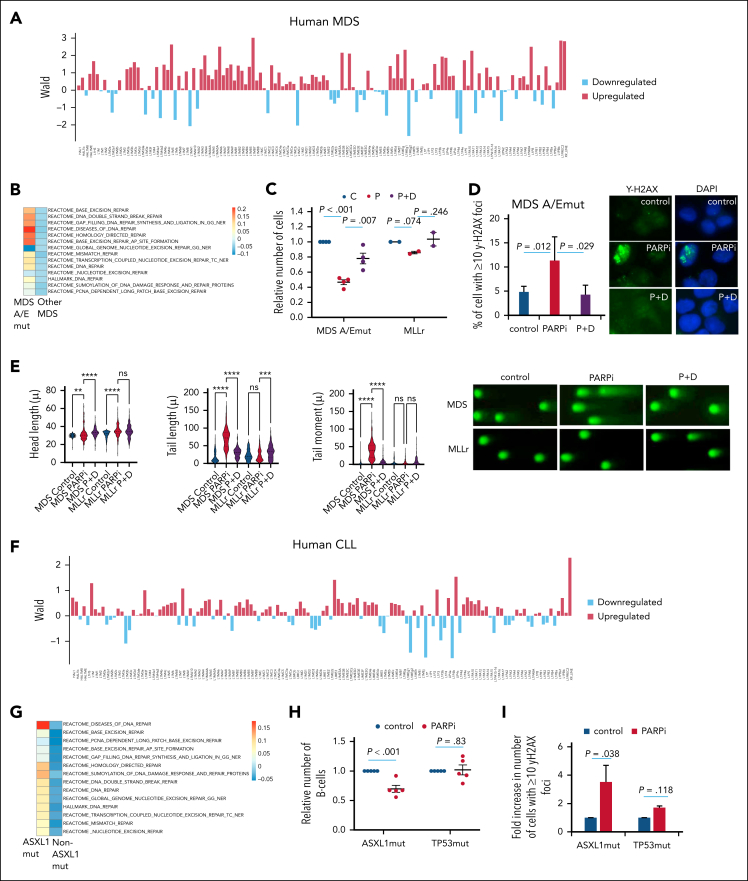

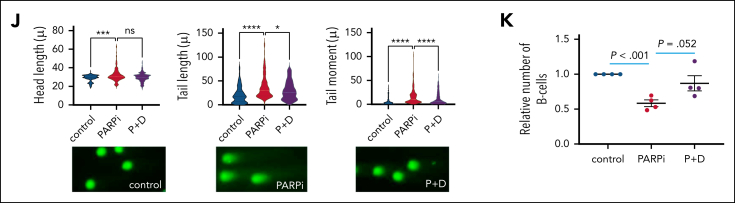
**Human myeloid malignancies with ASXL1/EZH2 and lymphoid CLL with ASXL1 mutations show reactivation of L1 TEs and are sensitive to P treatment.** (A) Wald statistics are shown for L1 TEs for a comparison between human MDS BM samples with ASXL1/EZH2 (n = 3) mutations and human MDS BM samples with other mutations (n = 109). (B) GSVA was performed on the whole human MDS BM data set (n = 112) and mean values for ASXL1/EZH2 mutant samples and samples with other mutations are shown in the heat map for DNA repair-related gene sets. (C) The relative number of cells for the indicated patient samples in indicated treatment groups are shown. MDS A/Emut, n = 4; MLLr, n = 2. (D) The bar charts (left) show the percentage of cells with ≥10 γH2AX foci after the indicated treatment for MDS A/Emut. Representative images (right) of γH2AX foci (green) and their merge with DAPI (blue) for the indicated treatments are shown. (E) Comet assay was performed after the indicated treatment on MDS A/Emut and MLLr cells and head length, tail length, and tail moment are shown in the violin plots (left). Example pictures for C and other treatments for the indicated cell types are shown (right). (F) Wald statistics are shown for L1 TEs for a comparison between human CLL samples with ASXL1 (n = 5) mutations and human CLL with other mutations (n = 161). (G) GSVA was performed on the whole human CLL data set (n = 166) and mean values for ASXL mutant samples and samples with other mutations are shown in the heat map for DNA repair-related gene sets. (H) The relative number of B cells of human CLL samples with the indicated mutations (n = 5 each) is shown after in vitro culture with or without P. (I) The fold increase in number of cells with ≥10 γH2AX foci is shown for the indicated human CLL cells with or without P treatment. (J) Comet assay was performed after the indicated treatment on human ASXL1-mutated CLL cells and head length, tail length, and tail moment are shown in the bar charts (top). Example pictures are shown (below). (K) The relative number of B cells of human CLL samples with ASXL1 mutations is shown with or without the indicated treatments (n = 4). Plots show mean ± SEM, ∗*P* < .05, ∗∗*P* < .01, ∗∗∗*P* < .001, ∗∗∗∗*P* < .001, unpaired *t* test. C, control; L1, LINE1; MDS A/Emut, human ASXL1/EZH2-mutated MDS cells; MLLr, MLL-rearranged AML; P, PARPi; P+D, PARPi + didanosine.

To examine whether similar findings in our mouse models can also be translated to human leukemia, we examined the TE expression profile of samples from patients with MDS carrying ASXL1/EZH2 mutations.^57^ In line with our mouse models, we observed (1) preferential upregulation of retroviral TEs, LINE1 (Figure 6A; supplemental Figure 6A-D), which is the only major active TEs that can elicit DDR in the human genome^24^^,^^25^; and (2) activation of DDR pathways in patient samples with Asxl1/Ezh2 mutations (Figure 6B). Consistently, at the functional level, these ASXL1/EZH2-mutated MDS samples were also sensitive to PARPi treatment (Figure 6C), which induced excessive γH2AX DNA damage foci (Figure 6D) and dsDNA breaks (Figure 6E; supplemental Figure 6E) in the PcG mutant but not in control MLL-rearranged samples. Comparable inhibitory effects were also observed using different PARPis, including veliparib and talazoparib (supplemental Figure 6F-I). Next, we performed similar analyses on samples from human patients with CLL with ASXL1 mutation compared with samples from those with non–ASXL1-mutated CLL reported in previous studies by us and others.^10^^,^^11^ In line with our findings in MDS samples, we observed activation of LINE1 (Figure 6F) and DDR pathways (Figure 6G) in ASXL1-mutated CLL. Importantly, cells from patients with ASXL1-mutated CLL were also sensitive to PARPi treatment that was otherwise ineffective on non–ASXL1 (TP53)-mutated CLL cells (Figure 6H). Moreover, PARPi treatment induced significant DNA damage in ASXL1-mutated CLL but not WT CLL (Figure 6I; supplemental Figure 6J). Consistently, the observed PARPi sensitivity as in our mouse models could also be partially reversed by RTis (Figure 6J-K; supplemental Figure 6K). Together with the mouse data, these findings consistently indicate that reactivation of TEs as a result of LoF mutation in PcG proteins provides a novel vulnerability for PARPi-induced synthetic lethal targeting of these cancers.

## Discussion

**Figure 7.**
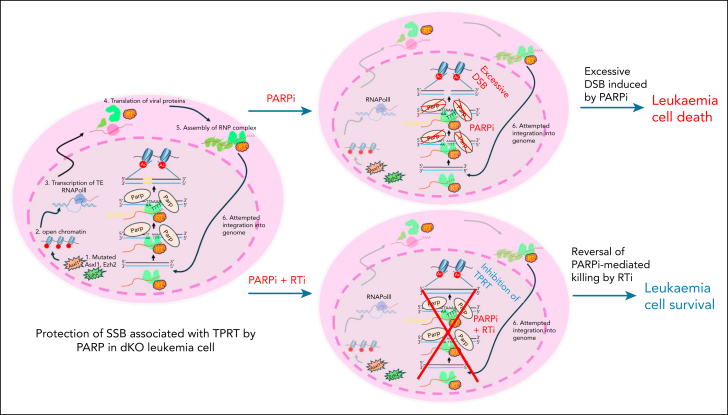
**Model describing the targeting of TPRT-induced DNA damage with PARPi.** An Asxl1/Ezh2 double-mutated (or KO) leukemia cell is depicted (left). Mutation/KO of epigenetic repressors, Asxl1 and Ezh2 (1), leads to chromatin opening; (2) which allows transcription of TEs (3). In the cytoplasm, translation of viral proteins (4) and assembly of RNP complexes (5) take place. Upon re-entry into the nucleus during DNA replication, viral RNA attempts to integrate via TPRT into the host genome (6). Briefly, viral proteins introduce a single-strand break (nick) in the host genome, creating ssDNA, which is protected by PARP proteins. Viral RNA binds to the ssDNA, which serve as a primer for the reverse transcriptase reaction to synthesize viral cDNA, which is then be integrated into the host genome. In the presence of PARPi, PARP proteins–mediated protection of the single-strand breaks/ssDNA is compromised, ultimately leading to excessive DSBs and leukemia cell death (top, right, +PARPi). In the presence of PARPi and RTi, viral RNA integration via TPRT is inhibited (bottom, right, +PARPi and RTi). RTi prevents the synthesis and integration of viral cDNA, thereby minimizing the genomic damage caused by reactivated TEs. As a result, leukemia cell may survive the PARPi treatment. cDNA, complementary DNA; DSB, DNA double-strand break; RNP, ribonucleoprotein.

In contrast to gain-of-function mutations, LoF mutations are typically intractable by classical small molecule inhibitor approaches. Synthetic lethality can be an ideal approach to target mutations leading to compromised DNA repair responses such as LoF BRCA mutations in ovarian and breast cancers.^58^^,^^59^ However, mutations affecting DDR genes are not common in many cancers including leukemias, which are frequently driven by mutations affecting transcriptional and epigenetic gene regulations.^40^^,^^56^^,^^60^ We and others have previously shown that oncogenic drivers can confer PARPi sensitivity to leukemia cells because of their impacts on HR (eg, AML1-ETO, promyelocytic leukemia-retinoic acid receptor alpha, ten-eleven translocation 2),^40^^,^^61^ or can be targeted when PARPis are used in combination with other therapeutics (eg, MLL fusions).^40^^,^^62^^,^^63^ In this study, we reveal a novel application of PARPi-induced synthetic lethality by targeting TPRT associated with reactivated TEs in leukemia driven by PcG LoF mutations (Figure 7), which is associated with very poor prognosis and do not have effective therapies. Although the ubiquitous and abundant nature of TEs present in cancer cells can have great potentials as molecular targets, very little progress has been made in deriving strategies considering TE reactivation for cancer therapeutics.^64^ To date, the only and most well-documented potential avenue is based on putative immunological responses triggered by reactivated TEs^28^, ^29^, ^30^, ^31^, ^32^, ^33^, ^34^^,^^65^^,^^66^; however, many patients with cancer still fail to mount proper immune responses because of cell intrinsic and/or extrinsic factors such as tumor microenvironment.^67^, ^68^, ^69^

After genetic losses of major epigenetic regulators commonly found in hematological malignancies, we showed that inactivation of PcG resulted in epigenetic reactivation of TEs and DNA damage in both mouse models and primary human patient samples. Strikingly, although reactivated TEs do not seem required for leukemic transformation, they create a vulnerability to PARPis. Consistently, PARPi treatment alone can significantly suppress leukemia cell growth and extend the disease latency. PARPi-induced DNA damage and selective killing can be partially blunted by RTis that suppress the formation of complementary DNA intermediate, key for TE life cycle PRC-mutated cells (Figure 7). The mechanism underlying the observed synthetic lethality by targeting specific functions of PARP in TPRT is intriguing and distinct from the classical PARPi sensitivity mediated by HR deficiency.^40^^,^^70^ Although future mechanistic and orthogonal studies (eg, CRISPR interference targeting specific classes of TE subfamilies, and integrase inhibitor studies) are needed to further pinpoint the subfamilies of TEs and dissect key molecular players mediating the PARPi phenotypes, this study sets the stage for a novel and broader approach of creating synthetic lethality for human cancers (Figure 7).

Evidently, the expression of TEs is tightly regulated by epigenetic (eg, PIWI-interacting RNA, DNA methylation, and histone modifications) and transcription (eg, KRAB-containing zinc-finger proteins) factors.^25^ Although spatial- and temporal-specific gene regulations are likely governed by a combination of transcriptional and epigenetic regulators, it is tempting to speculate potential utility of PARPis in cancers carrying epigenetic mutations (eg, DNMT, SETDB1, and PRC) that may lead to similar derepression of TEs. Future studies examining the different cell type–specific mechanisms of epigenetic regulations of TEs will shed light on this issue and help to further broaden the application of PARPi-induced synthetic lethality in other cancers.

Conflict-of-interest disclosure: The authors declare no competing financial interests.

## Supplementary Material

Supplemental Figures, Tables, Methods, and References
