# Supplementary material for: Transposable elements as novel therapeutic targets for PARPi-induced synthetic lethality in PcG-mutated blood cancer
Source: Blood. 2025 Jul 8;146(11):1314–30. doi: 10.1182/blood.2025028560 (PMC12824672; doi:10.1182/blood.2025028560)
Supplement: Supplemental Figures, Tables, Methods, and References [file BLOOD_BLD-2025-028560-mmc1.pdf]

Supplementary Figure S1

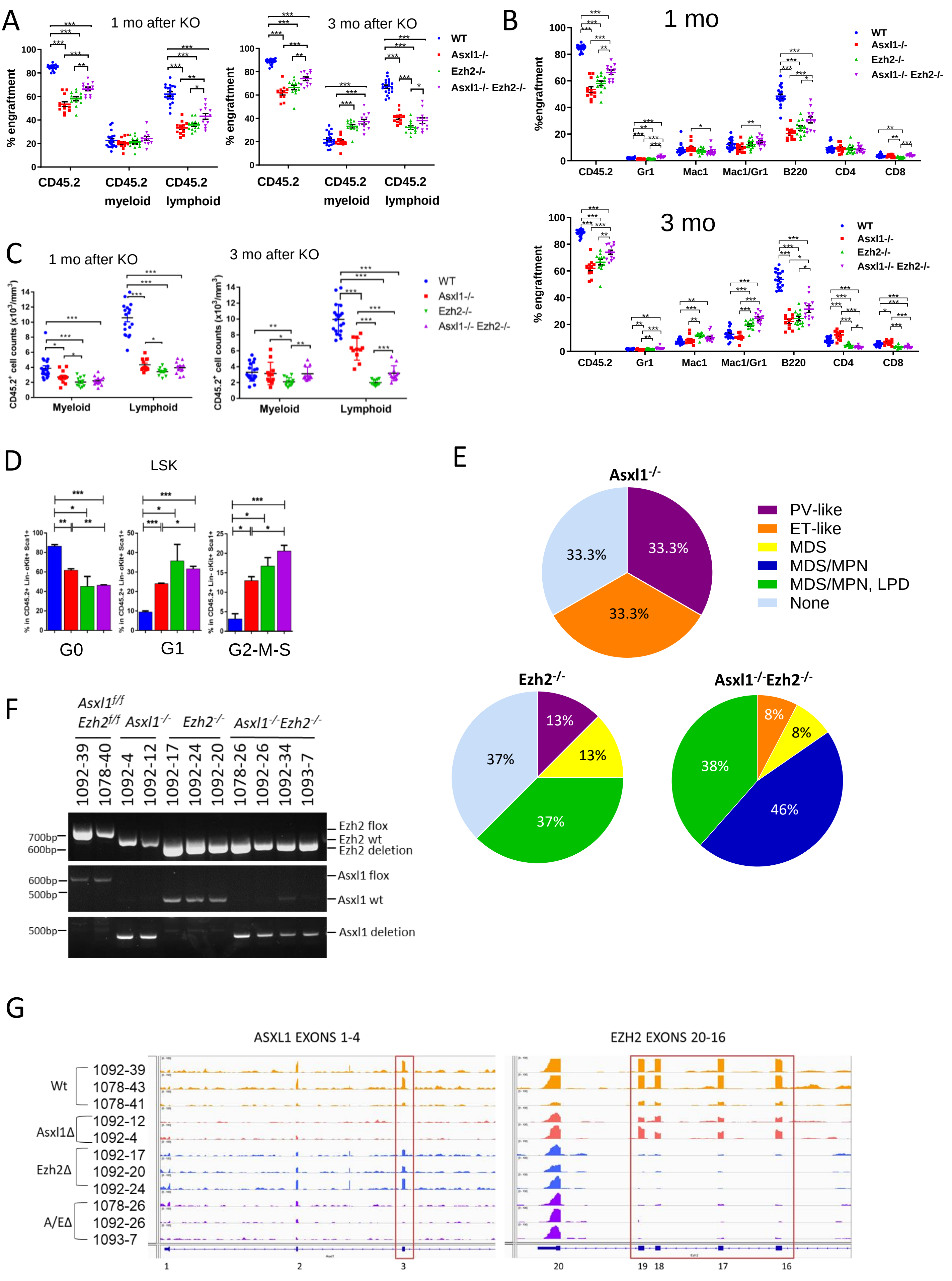

Supplementary Figure S2

A

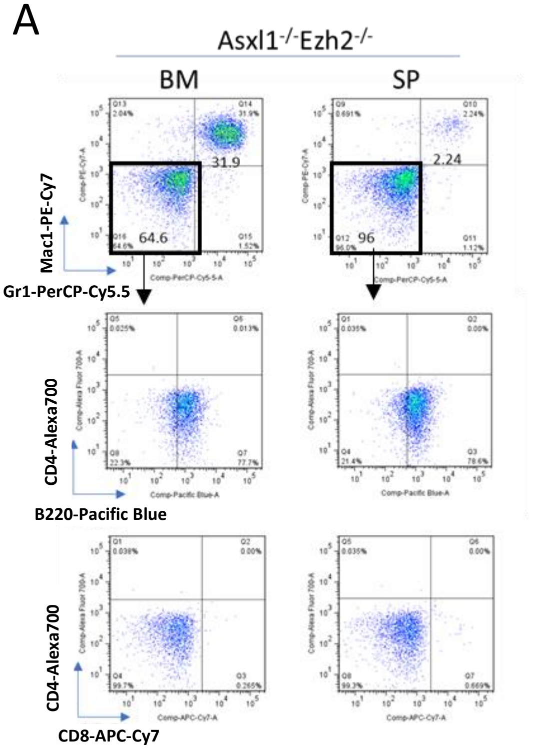

B

WT vs Asxl1-/-Ezh2-/-

	ID	Name	Source	pValue	FDR B&H	FDR B&Y	Bonferroni	Genes from Input	Genes in Annotation
1	GSM854229 500	B cells, B1a.Sp, CD19+ CD45R+ IgM++ AA4.1- CD23- CD43+ CD5+, Spleen, avg-3	Immgen.org, GSE15907	3.108E-145	1.545E-141	1.404E-140	1.545E-141	182	347
2	GSM538225 500	B cells, B1a.Sp, IgD+ IgM+ CD45R+ CD24+ CD19+ AA4.1- CD23- CD21/35-, Spleen, avg-3	Immgen.org, GSE15907	3.809E-129	9.466E-126	8.603E-125	1.893E-125	169	341

WT vs Ezh2-/-

	ID	Name	Source	pValue	FDR B&H	FDR B&Y	Bonferroni	Genes from Input	Genes in Annotation
1	GSM854229 500	B cells, B1a.Sp, CD19+ CD45R+ IgM++ AA4.1- CD23- CD43+ CD5+, Spleen, avg-3	Immgen.org, GSE15907	5.068E-48	2.102E-44	1.872E-43	2.102E-44	56	347
2	GSM538225 500	B cells, B1a.Sp, IgD+ IgM+ CD45R+ CD24+ CD19+ AA4.1- CD23- CD21/35-, Spleen, avg-3	Immgen.org, GSE15907	1.107E-40	2.295E-37	2.044E-36	4.590E-37	50	341

C

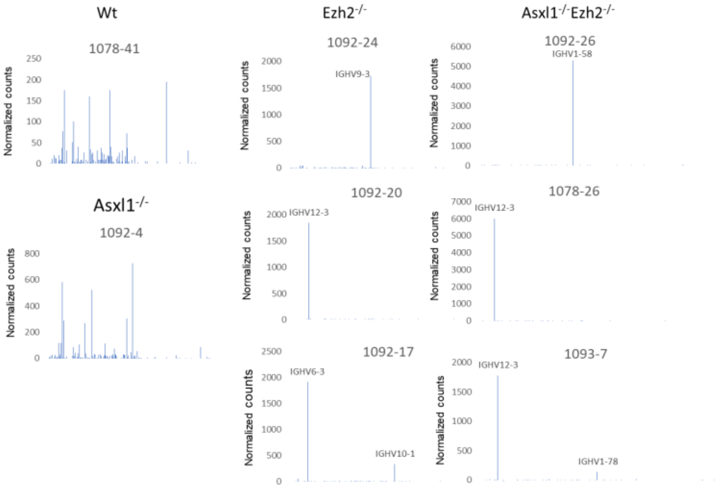

D

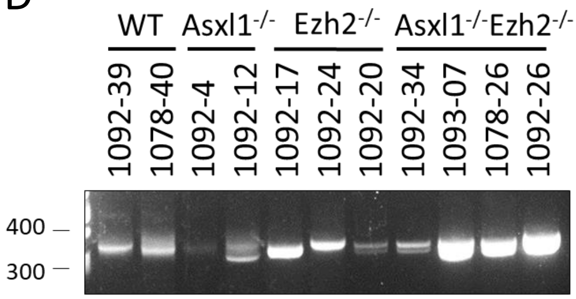

E

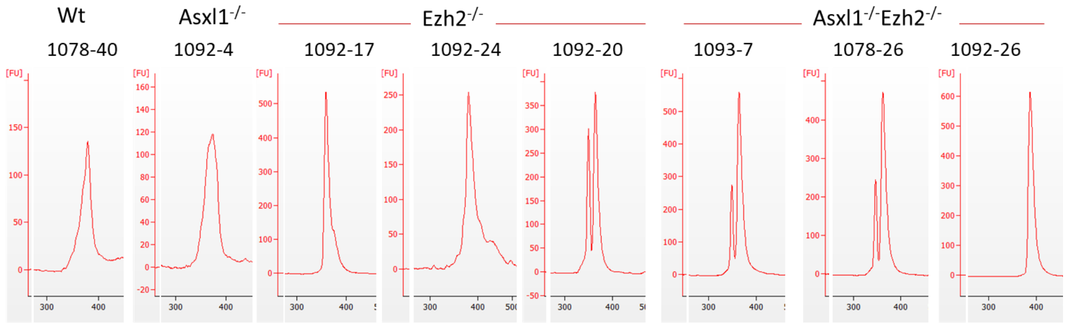

F

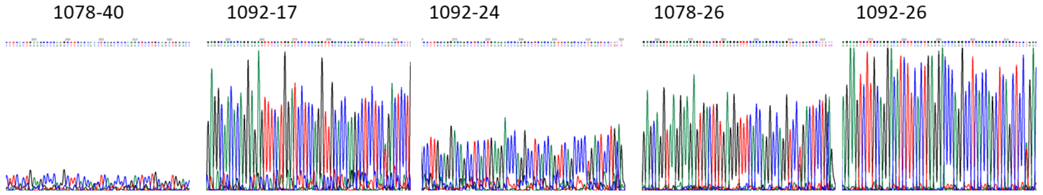

G

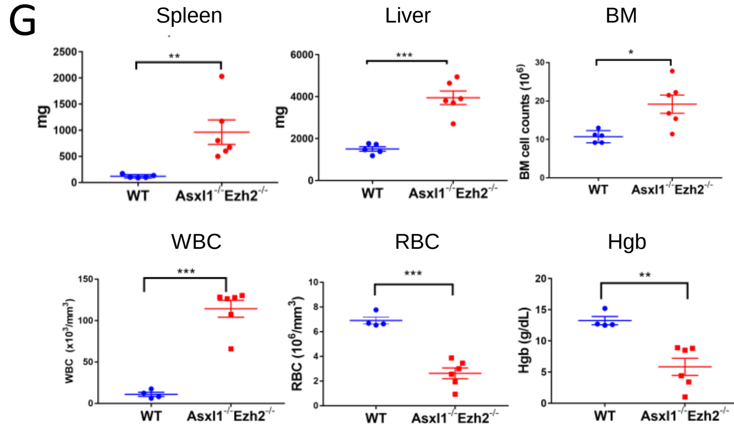

H

Transfer	Donor	ID	Clone	VH	D	JH	Mutation	HCDR3
2nd	1078-26	1281-1	Mono	V12-3	D4-1	JH1	(U)	A G D I G G Y W Y F D V
2nd	1092-26	1298-1	Mono	V1-58	D2-5	JH4	(U)	A R S D Y S N Y V S Y Y Y A M D Y
2nd	1092-26	1298-2	Mono	V1-58	D2-5	JH4	(U)	A R S D Y S N Y V S Y Y Y A M D Y
3rd	1298-1	1430-9	Mono	V1-58	D2-5	JH4	(U)	A R S D Y S N Y V S Y Y Y A M D Y
3rd	1298-1	1430-10	Mono	V1-58	D2-5	JH4	(U)	A R S D Y S N Y V S Y Y Y A M D Y
3rd	1298-1	1430-11	Mono	V1-58	D2-5	JH4	(U)	A R S D Y S N Y V S Y Y Y A M D Y

Analysis of IGHVDJ from splenocytes of Asxl1$^{-/-}$Ezh2$^{-/-}$ CLL mice.
Mono (Mono-clone), U (unmuted IGHV), M (mutated IGHV)
Heavy chain complementary determining region (HCDR3)

I

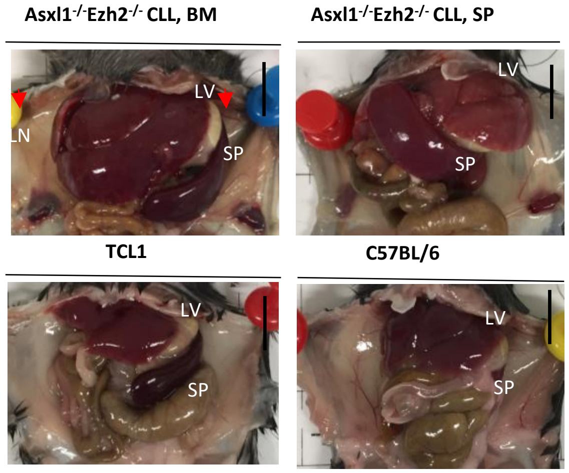

J

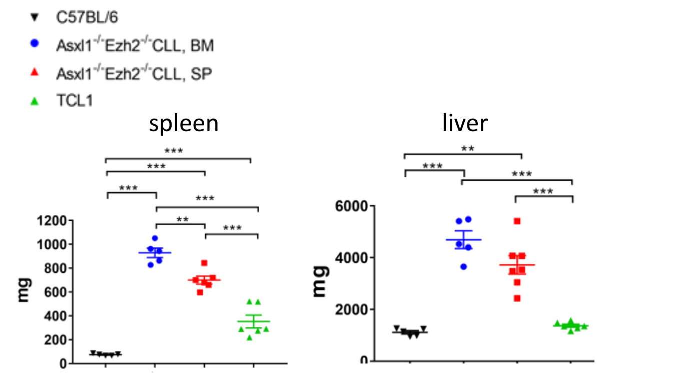

K

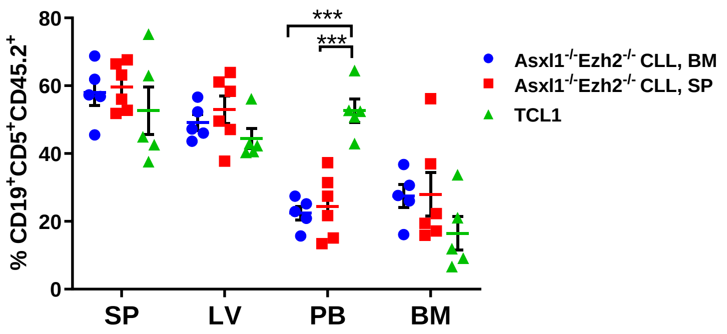

L

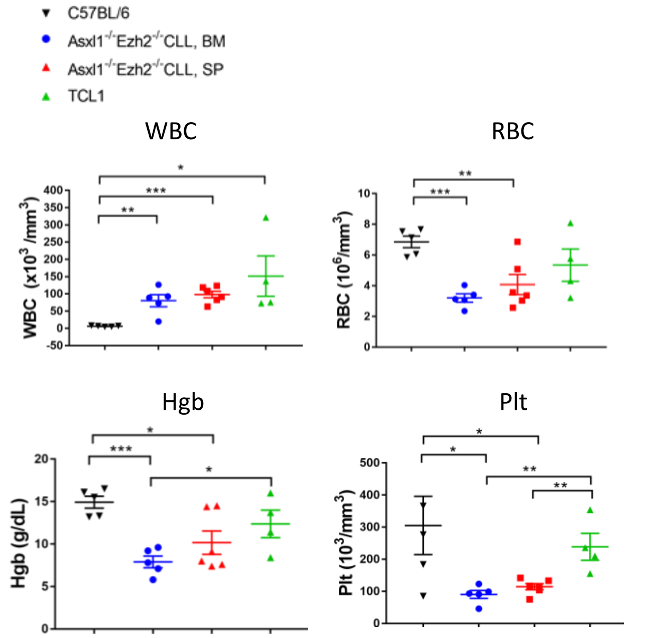

M

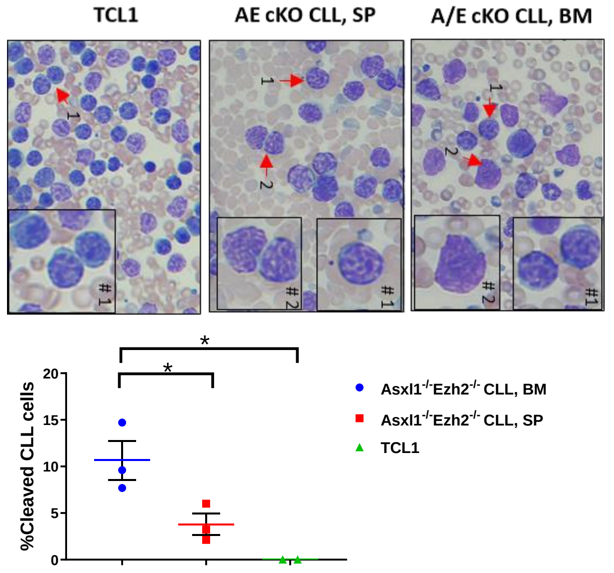

Supplementary Figure S3

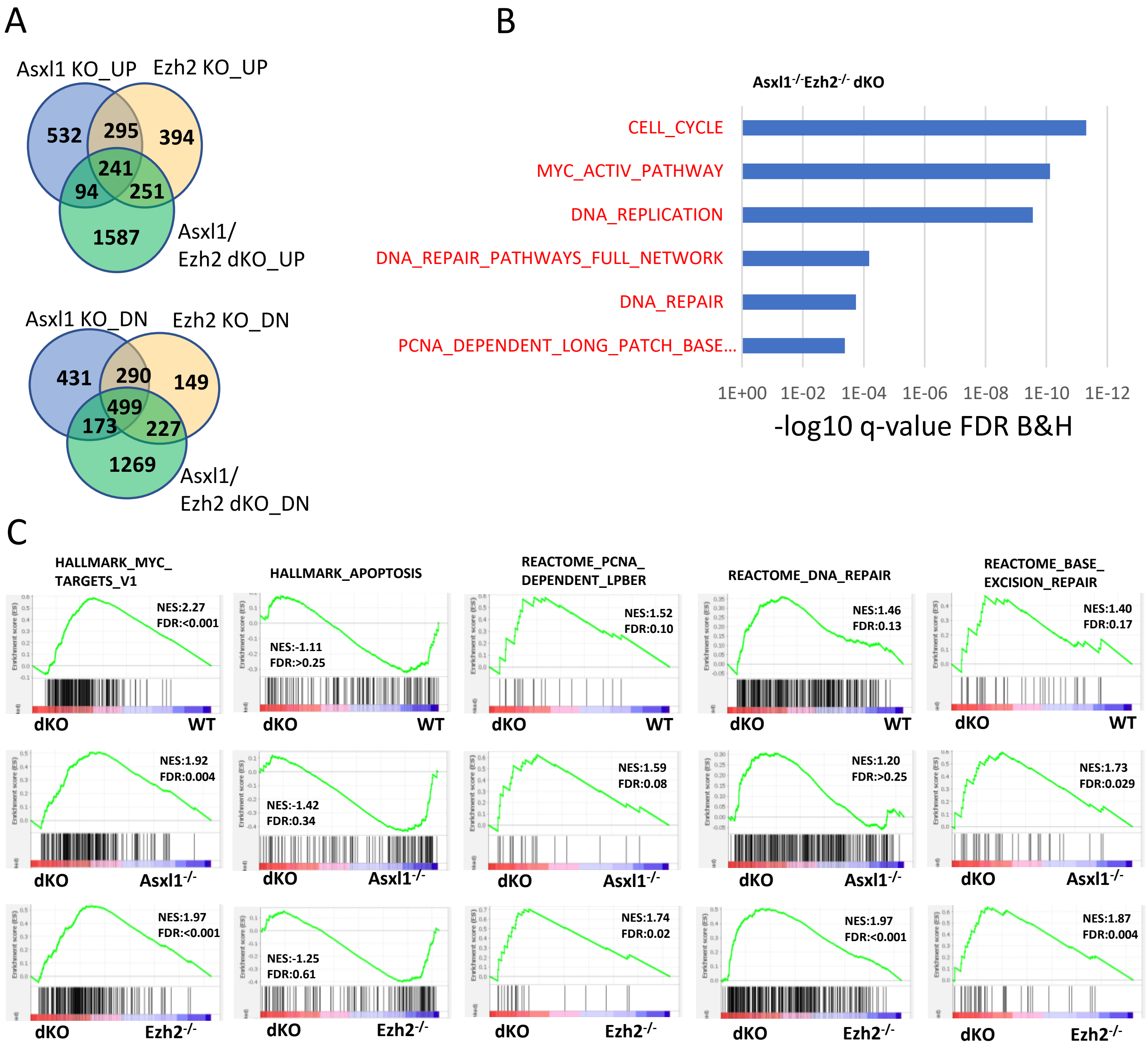

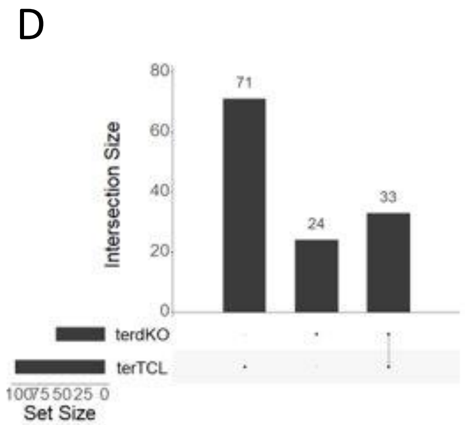

E

	WT v dKO BM		WT v dKO CD19+		TCL1 v dKO CD19+	
**mgi_symbol**	**log2FC**	**padj**	**log2FC**	**Padj**	**log2FC**	**padj**
Brca1	0.108	8.36E-01	2.230	**5.83E-22**	0.799	**2.49E-04**
Fbxo32	1.269	**3.89E-02**	-0.888	**4.91E-03**	-1.048	**2.53E-03**
Cdkn1a	-1.324	**1.68E-02**	5.122	**1.15E-60**	1.144	**8.36E-08**
Rad51	0.074	9.14E-01	1.722	**8.99E-13**	0.533	**3.66E-02**
Mad2l2	0.919	**6.53E-02**	0.529	**4.99E-02**	0.364	2.81E-01
Carm1	-0.092	8.78E-01	0.740	5.32E-04	-0.030	9.44E-01

F

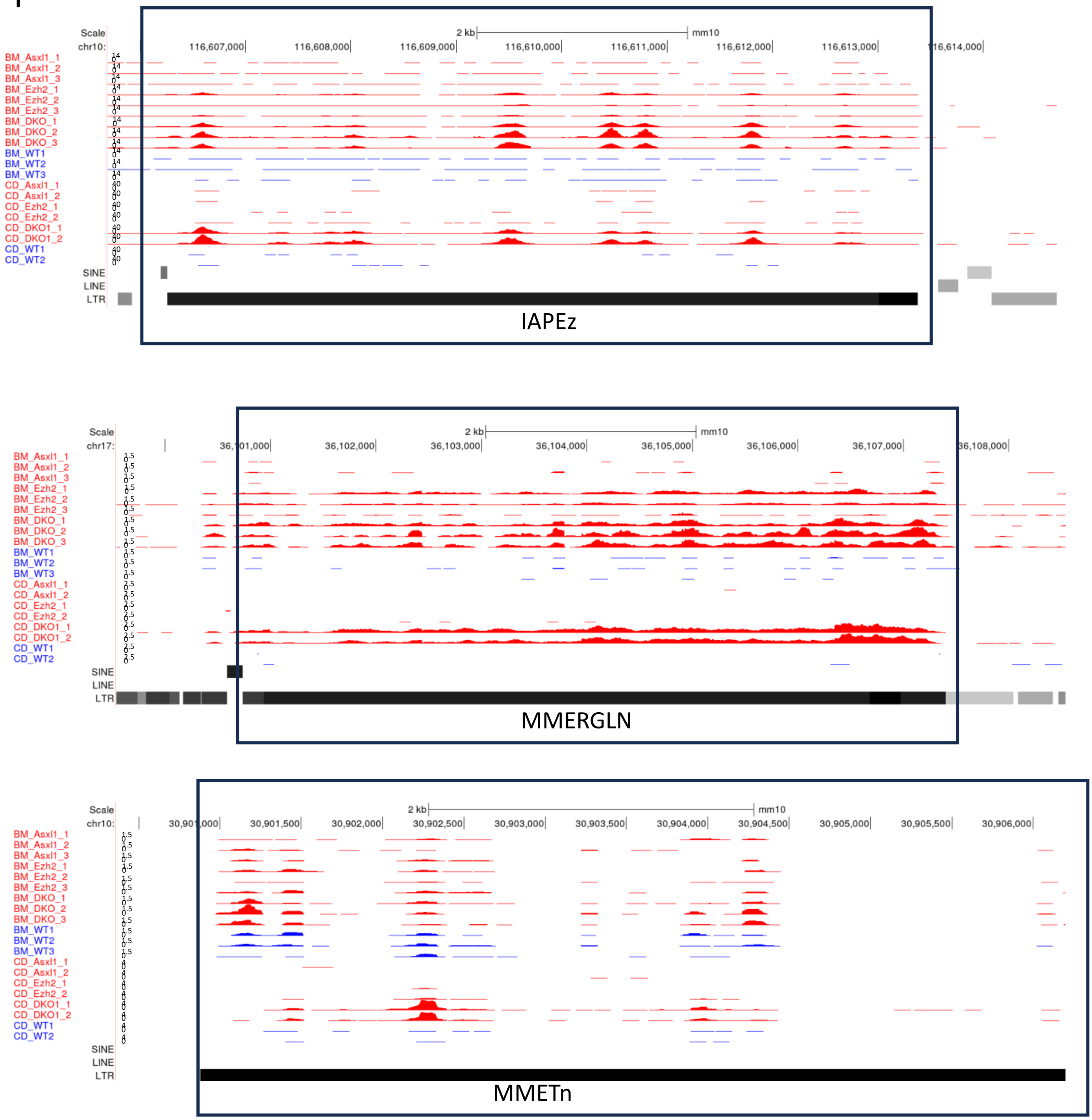
Scale
chr10:
2 kb
mm10
116,607,000
116,608,000
116,609,000
116,610,000
116,611,000
116,612,000
116,613,000
116,614,000
BM_Asxl1_1
BM_Asxl1_2
BM_Asxl1_3
BM_Ezh2_1
BM_Ezh2_2
BM_Ezh2_3
BM_DKO_1
BM_DKO_2
BM_DKO_3
BM_WT1
BM_WT2
BM_WT3
CD_Asxl1_1
CD_Asxl1_2
CD_Ezh2_1
CD_Ezh2_2
CD_DKO1_1
CD_DKO1_2
CD_WT1
CD_WT2
SINE
LINE
LTR
IAPEz
Scale
chr17:
2 kb
mm10
36,102,000
36,103,000
36,104,000
36,105,000
36,106,000
36,107,000
36,108,000
MMERGLN
Scale
chr10:
2 kb
mm10
30,901,500
30,902,000
30,902,500
30,903,000
30,903,500
30,904,000
30,904,500
30,905,000
30,905,500
30,906,000
MMETn

G

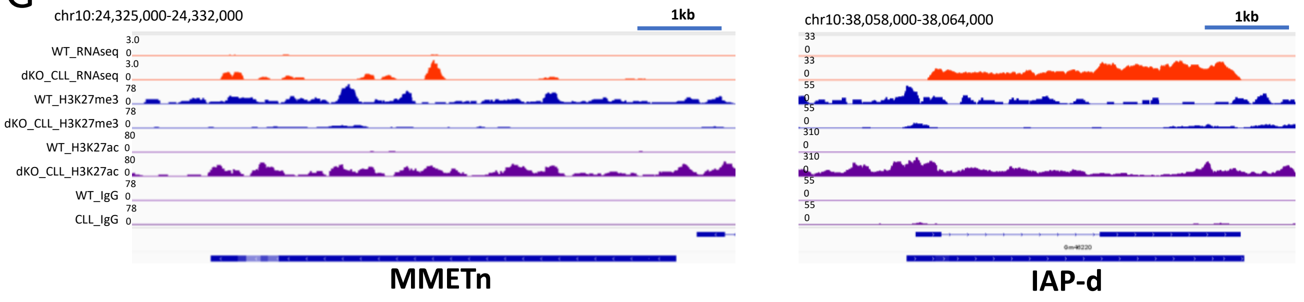
chr10:24,325,000-24,332,000
1kb
WT_RNAseq
dKO_CLL_RNAseq
WT_H3K27me3
dKO_CLL_H3K27me3
WT_H3K27ac
dKO_CLL_H3K27ac
WT_IgG
CLL_IgG
MMETn
chr10:38,058,000-38,064,000
1kb
IAP-d

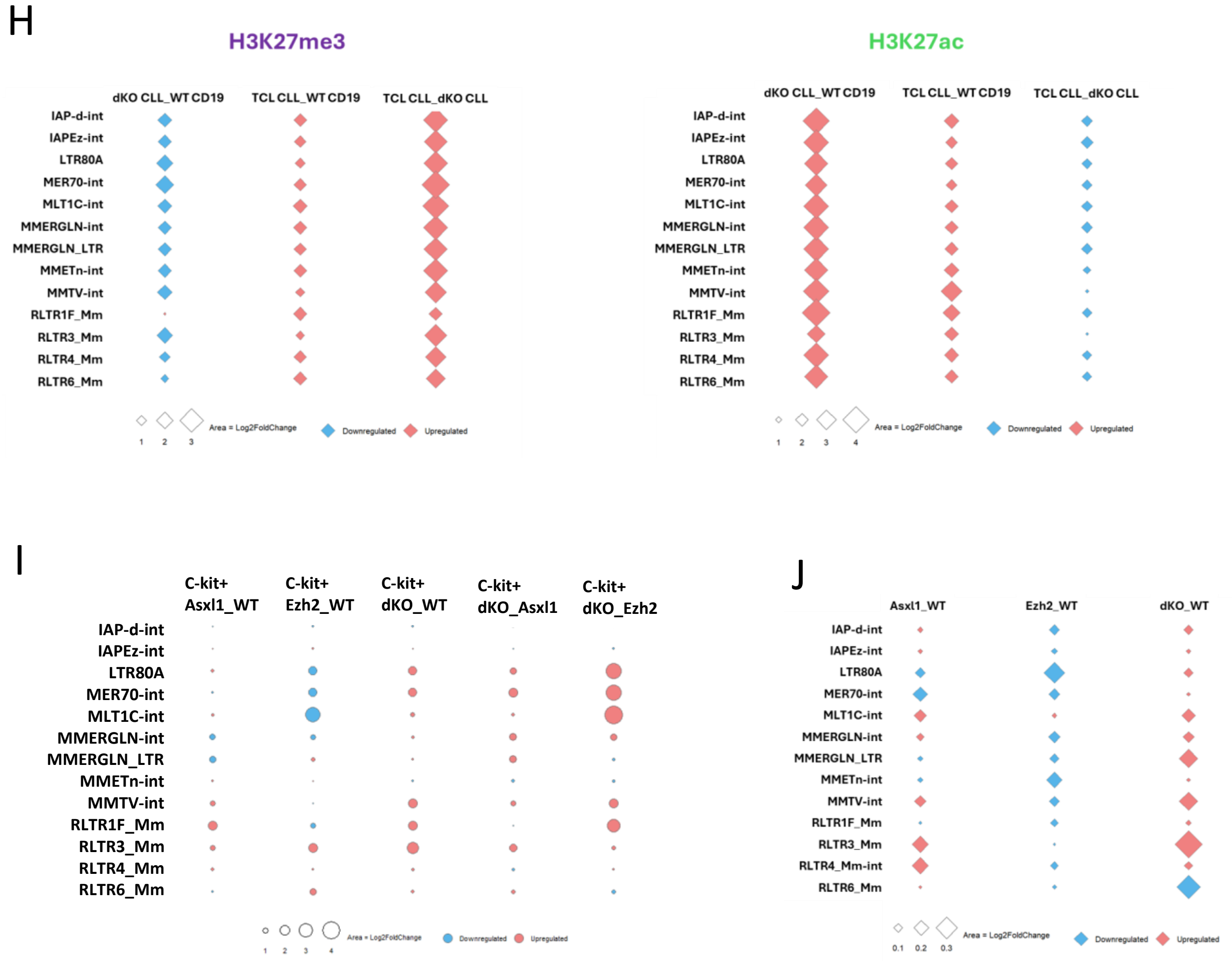

H
H3K27me3
dKO CLL_WT CD19
TCL CLL_WT CD19
TCL CLL_dKO CLL
IAP-d-int
IAPEz-int
LTR80A
MER70-int
MLT1C-int
MMERGLN-int
MMERGLN_LTR
MMETn-int
MMTV-int
RLTR1F_Mm
RLTR3_Mm
RLTR4_Mm
RLTR6_Mm
1 2 3
Area = Log2FoldChange
Downregulated
Upregulated
H3K27ac
dKO CLL_WT CD19
TCL CLL_WT CD19
TCL CLL_dKO CLL
IAP-d-int
IAPEz-int
LTR80A
MER70-int
MLT1C-int
MMERGLN-int
MMERGLN_LTR
MMETn-int
MMTV-int
RLTR1F_Mm
RLTR3_Mm
RLTR4_Mm
RLTR6_Mm
1 2 3 4
Area = Log2FoldChange
Downregulated
Upregulated
I
C-kit+ Asxl1_WT
C-kit+ Ezh2_WT
C-kit+ dKO_WT
C-kit+ dKO_Asxl1
C-kit+ dKO_Ezh2
IAP-d-int
IAPEz-int
LTR80A
MER70-int
MLT1C-int
MMERGLN-int
MMERGLN_LTR
MMETn-int
MMTV-int
RLTR1F_Mm
RLTR3_Mm
RLTR4_Mm
RLTR6_Mm
1 2 3 4
Area = Log2FoldChange
Downregulated
Upregulated
J
Asxl1_WT
Ezh2_WT
dKO_WT
IAP-d-int
IAPEz-int
LTR80A
MER70-int
MLT1C-int
MMERGLN-int
MMERGLN_LTR
MMETn-int
MMTV-int
RLTR1F_Mm
RLTR3_Mm
RLTR4_Mm-int
RLTR6_Mm
0.1 0.2 0.3
Area = Log2FoldChange
Downregulated
Upregulated

Supplementary Figure S4

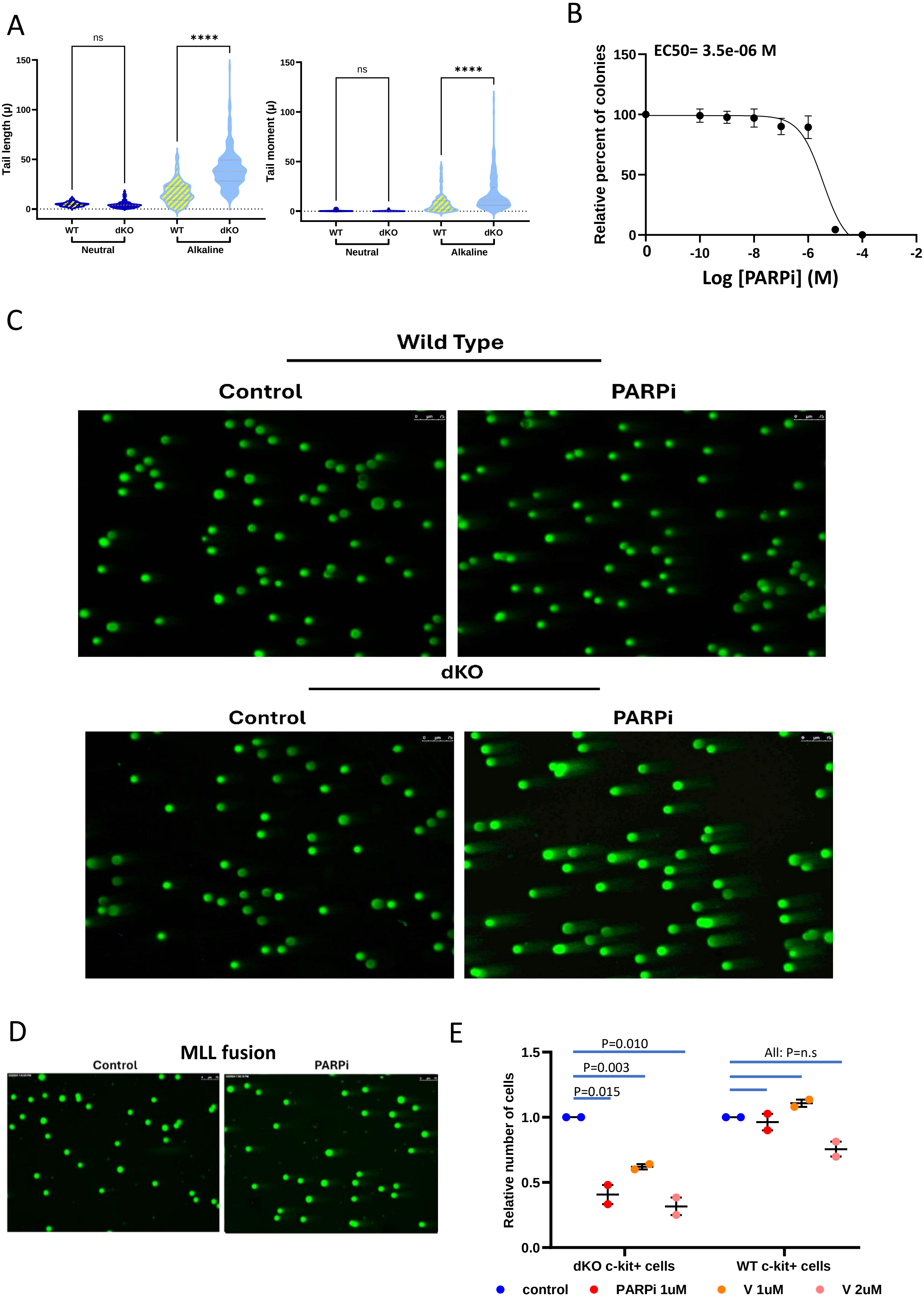

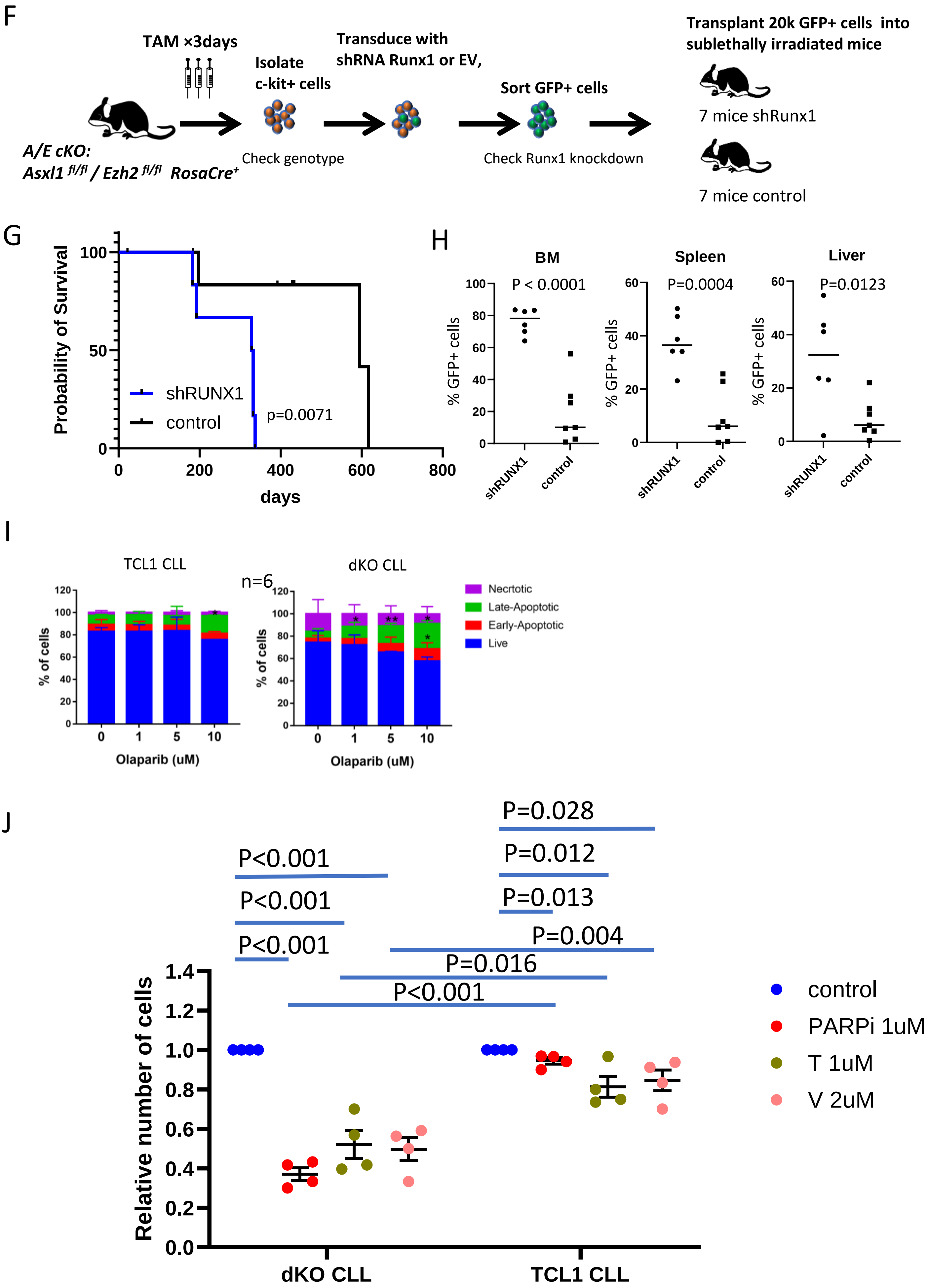
F
TAM ×3days
Isolate
c-kit+ cells
Transduce with
shRNA Runx1 or EV,
Sort GFP+ cells
Transplant 20k GFP+ cells into
sublethally irradiated mice
A/E cKO:
Asxl1 fl/fl / Ezh2 fl/fl RosaCre+
Check genotype
Check Runx1 knockdown
7 mice shRunx1
7 mice control
G
Probability of Survival
shRUNX1
control
p=0.0071
days
H
BM
Spleen
Liver
P < 0.0001
P=0.0004
P=0.0123
% GFP+ cells
shRUNX1
control
I
TCL1 CLL
dKO CLL
n=6
% of cells
Olaparib (uM)
Necrtotic
Late-Apoptotic
Early-Apoptotic
Live
J
P=0.028
P=0.012
P=0.013
P=0.004
P=0.016
P<0.001
P<0.001
P<0.001
P<0.001
Relative number of cells
dKO CLL
TCL1 CLL
control
PARPi 1uM
T 1uM
V 2uM

K

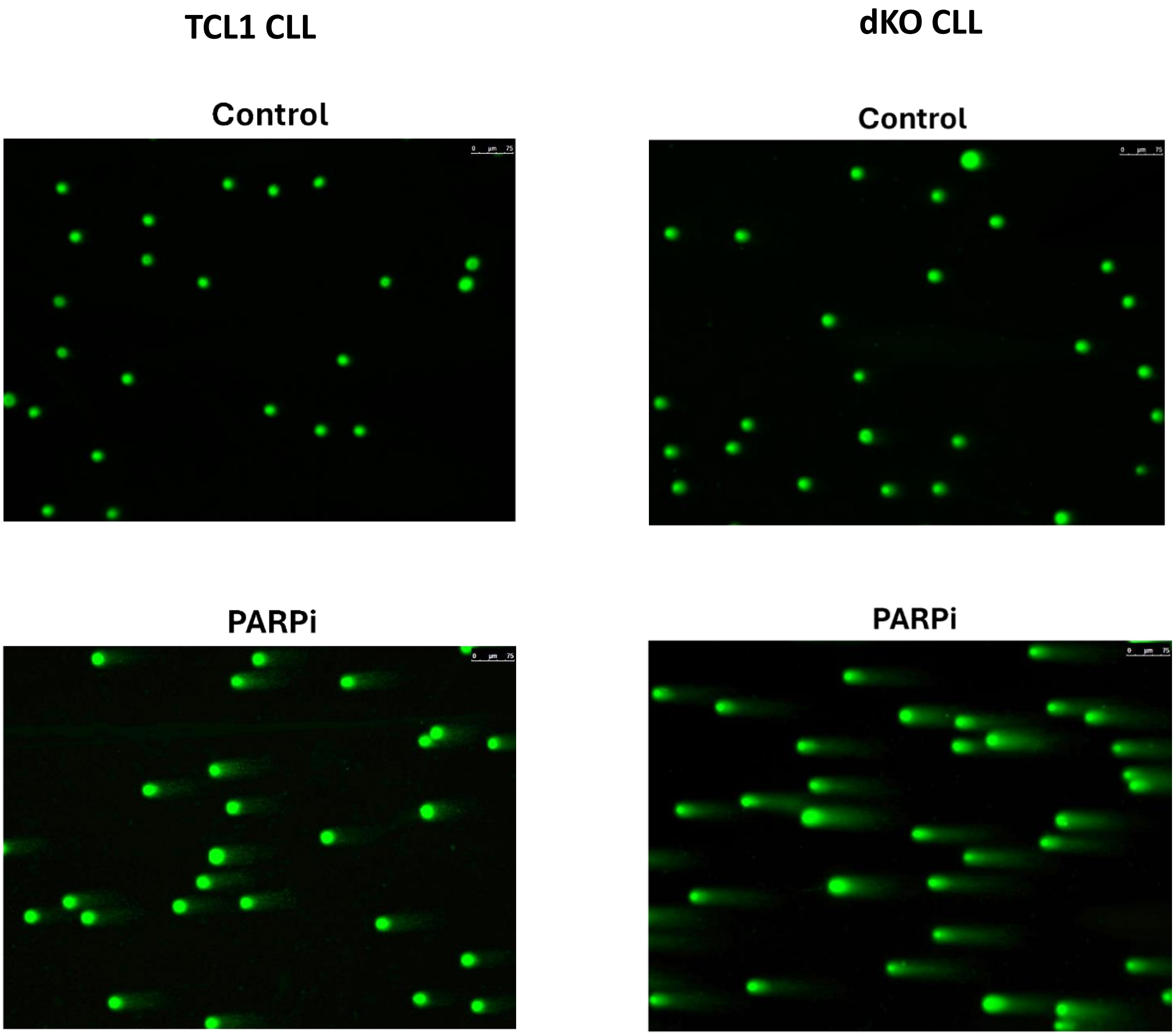
TCL1 CLL
dKO CLL
Control
Control
PARPi
PARPi

L

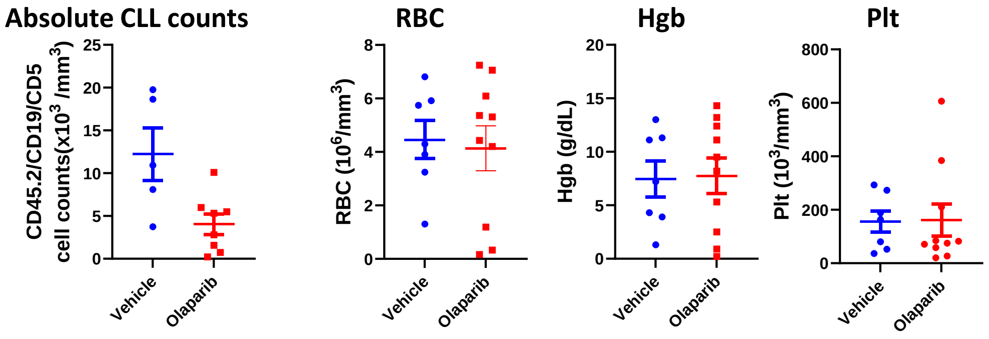
Absolute CLL counts
CD45.2/CD19/CD5 cell counts(x10^3 /mm^3)
25
20
15
10
5
0
Vehicle
Olaparib
RBC
RBC (10^6/mm^3)
8
6
4
2
0
Vehicle
Olaparib
Hgb
Hgb (g/dL)
20
15
10
5
0
Vehicle
Olaparib
Plt
Plt (10^3/mm^3)
800
600
400
200
0
Vehicle
Olaparib

Supplementary Figure S5

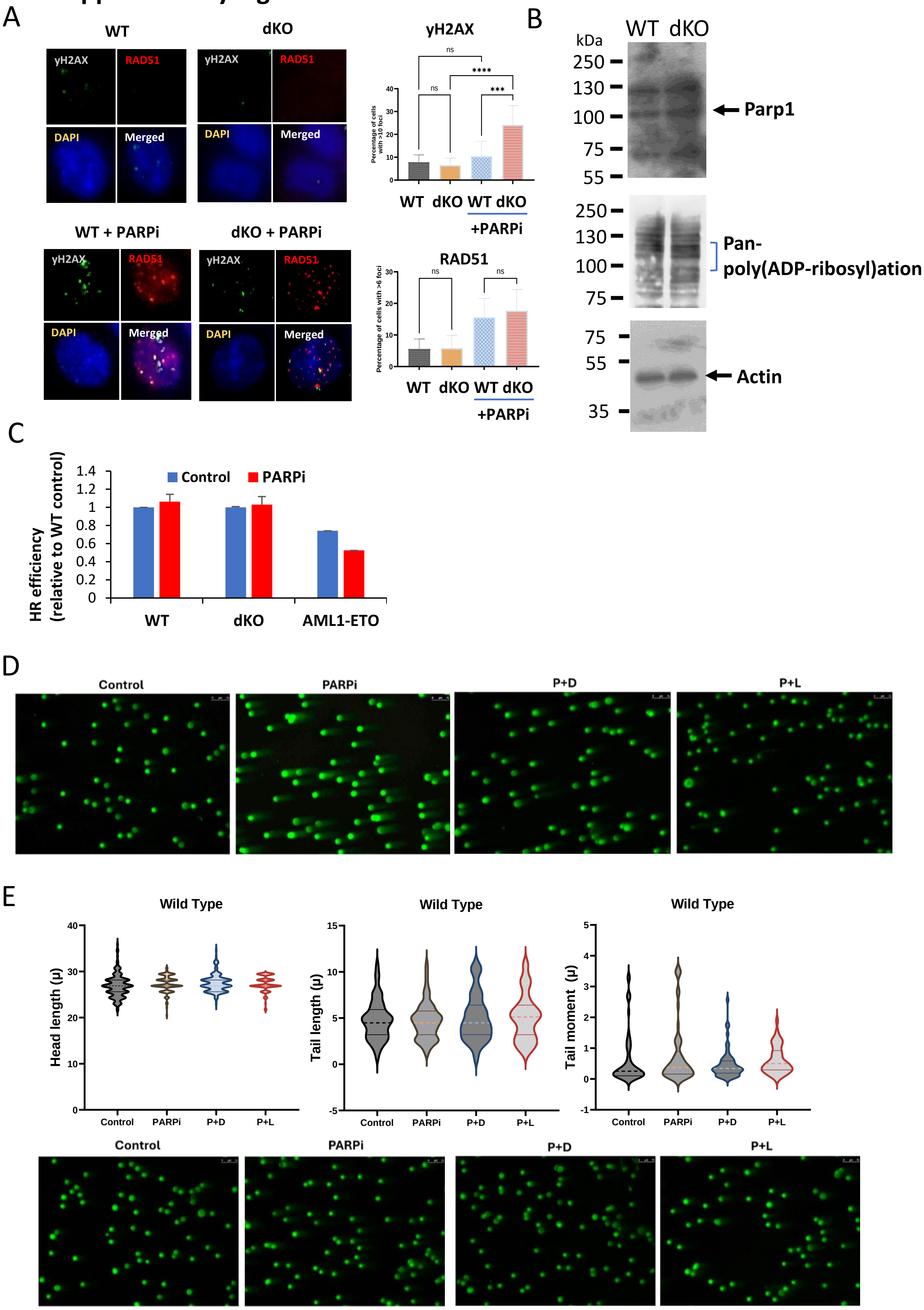

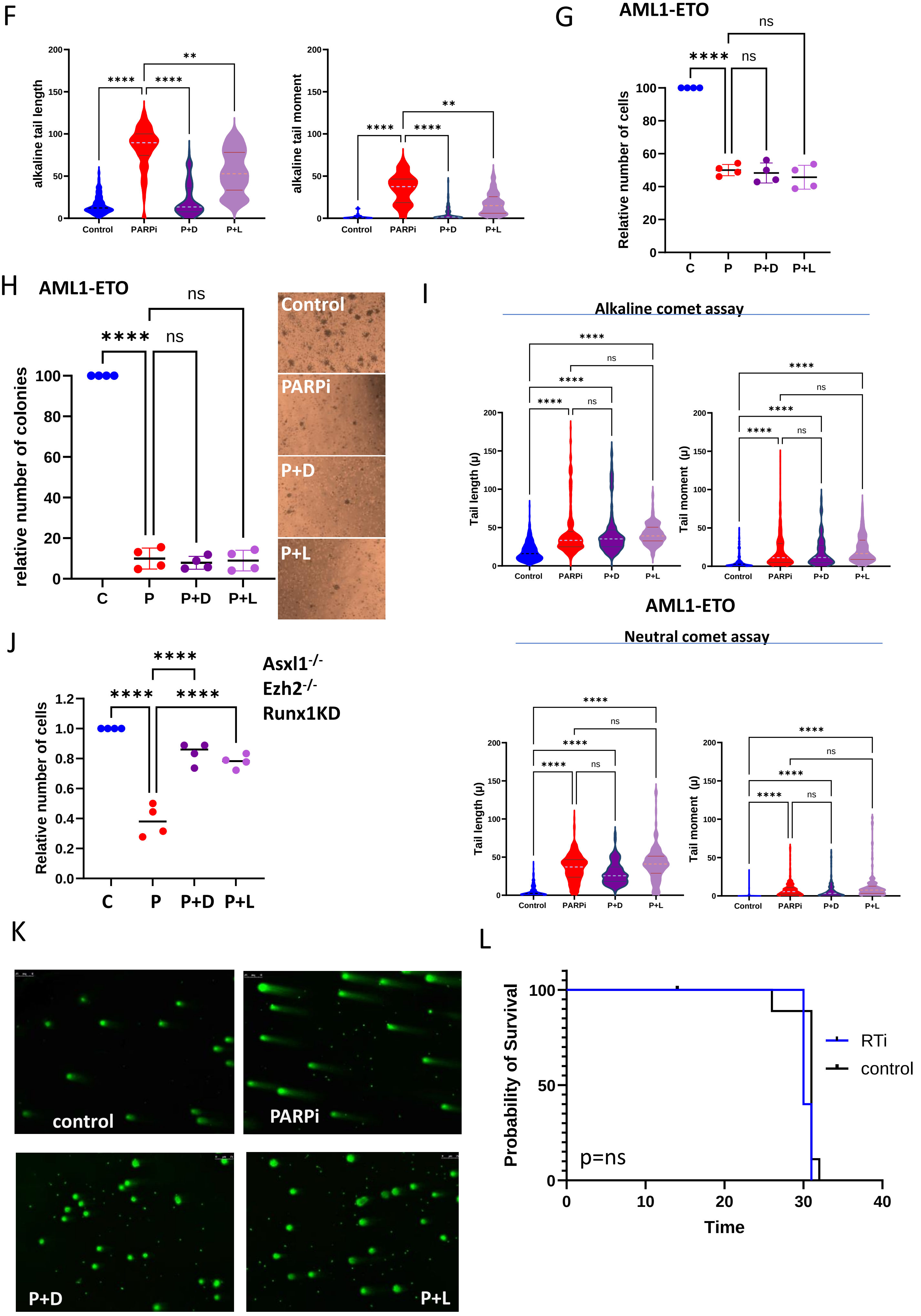

F
alkaline tail length
alkaline tail moment
Control
PARPi
P+D
P+L
****
**
G
AML1-ETO
Relative number of cells
ns
C
P
P+D
P+L
H
AML1-ETO
relative number of colonies
Control
PARPi
P+D
P+L
I
Alkaline comet assay
Tail length (μ)
Tail moment (μ)
AML1-ETO
Neutral comet assay
J
Asxl1-/-
Ezh2-/-
Runx1KD
Relative number of cells
K
control
PARPi
P+D
P+L
L
Probability of Survival
RTi
control
p=ns
Time

Supplementary Figure S6

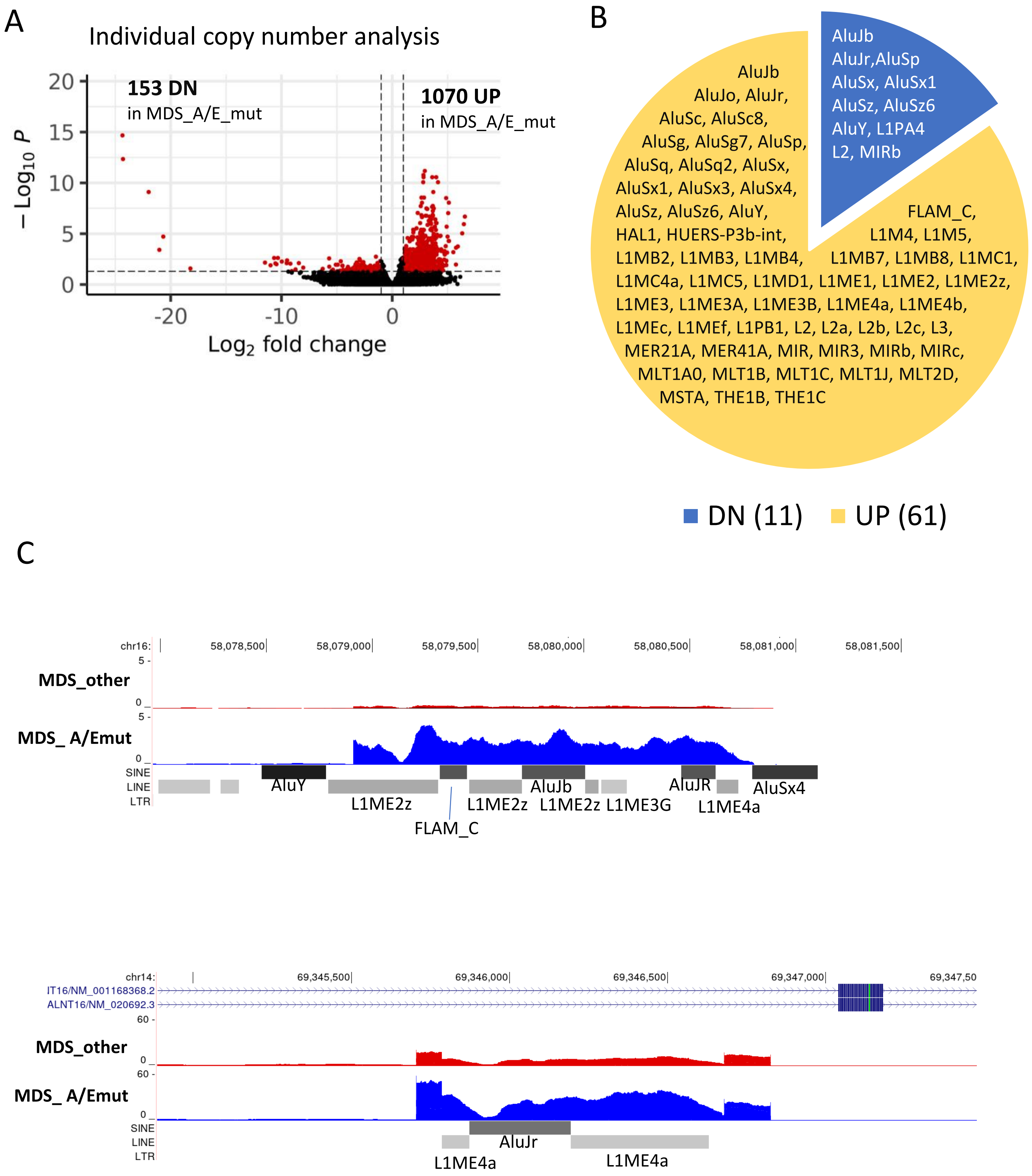

Supplementary Figure S6

D

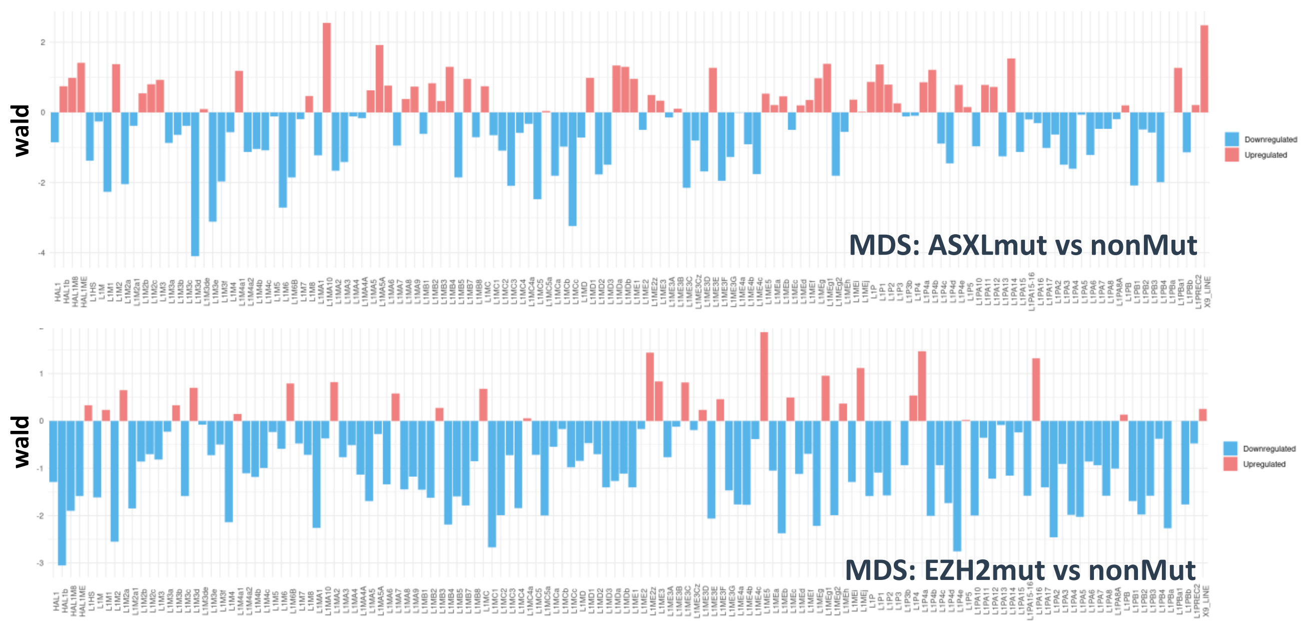

E

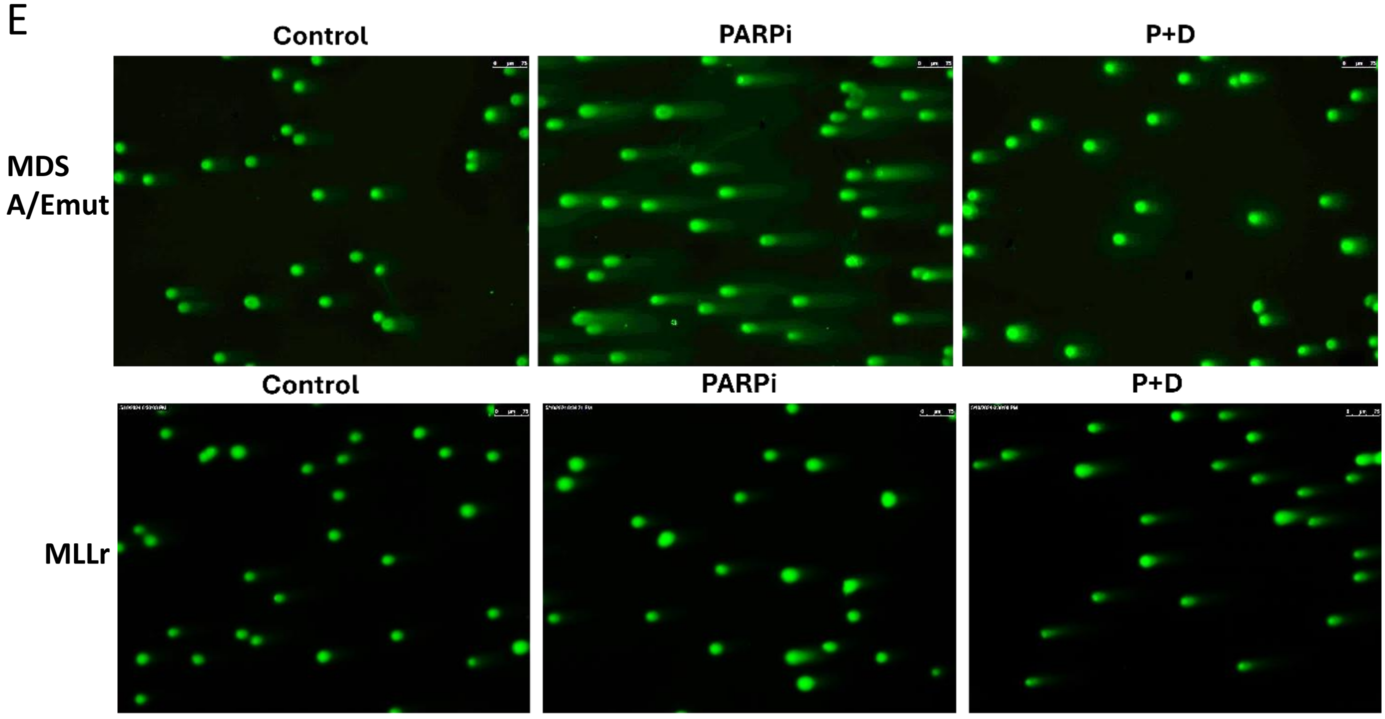

F

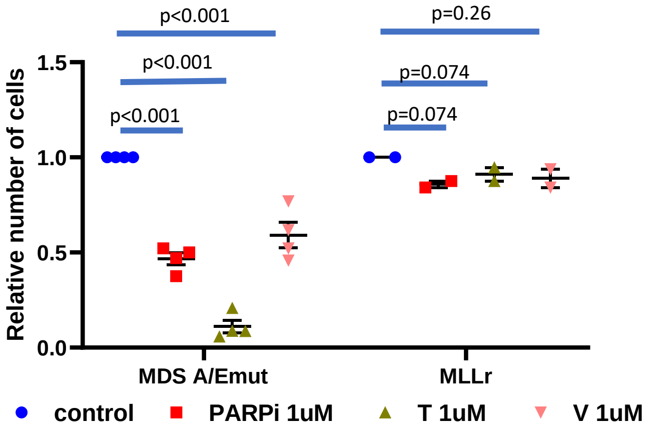

G

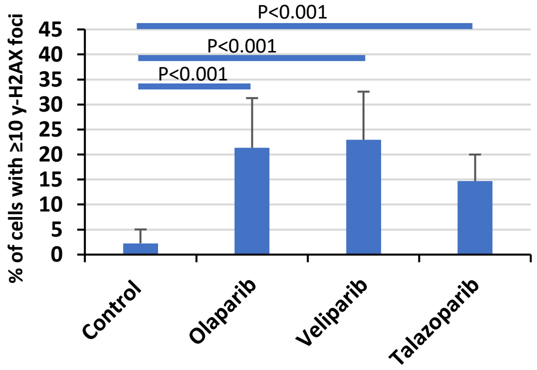

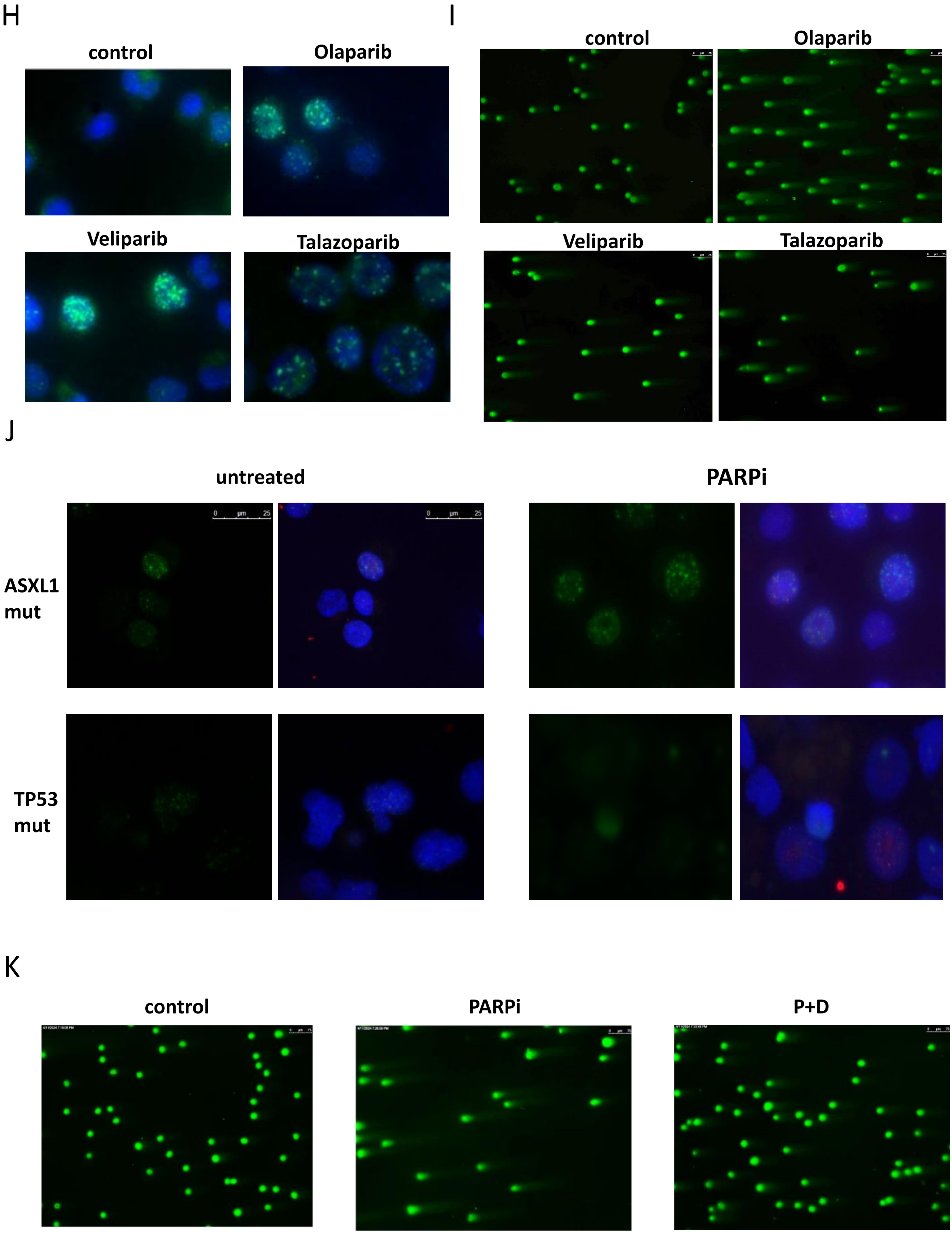
H
control
Olaparib
Veliparib
Talazoparib
I
control
Olaparib
Veliparib
Talazoparib
J
untreated
PARPi
ASXL1 mut
TP53 mut
0 μm 25
0 μm 25
K
control
PARPi
P+D

Supplementary Table S1

List of antibodies

Antibody	Supplier	Catalog NO	Application	Dillution
Phospho-yH2AX (ser139)	Upstate /Merck	05-636	Immunofluorescence	1 in 200 to 1 in 500
Alexa-488 Donkey anti-mouse IgG	Jackson ImmunoResearch	715-545-150	Immunofluorescence	1 in 400
RAD51	Merck Millipore	ABE257	Immunofluorescence	1 in 333
Cy™3 AffiniPure goat anti-rabbit IgG	Jackson ImmunoResearch	111-165-144	Immunofluorescence	1 in 333
DAPI	Merck	D9542-1MG	Immunofluorescence	1 in 333
APC-CD45.2	Biolegend	109814	FACS (General staining)	1 in 400
FITC-CD45.1	Biolegend	110706	FACS (General staining)	1 in 400
PerCP-Cy5.5-Gr1	Biolegend	108428	FACS (General staining)	1 in 400
PE-Cy7-Mac1	Biolegend	101216	FACS (General staining)	1 in 400
Pacific Blue-B220	Biolegend	103227	FACS (General staining)	1 in 400
Alexa700-CD4	Biolegend	100430	FACS (General staining)	1 in 400
APC-Cy7-CD8α	Biolegend	100714	FACS (General staining)	1 in 400
PE-c-kit	Biolegend	105808	FACS (General staining)	1 in 400
APC-Cy7-CD19	Biolegend	115519	FACS (CLL staining)	1 in 400
Alexa700-CD5	Biolegend	100635	FACS (CLL staining)	1 in 400
FITC-CD43	Biolegend	553270	FACS (CLL staining)	1 in 400
PerCP-Cy5-IgM	Biolegend	406512	FACS (CLL staining)	1 in 400
FITC-kappa	Biolegend	40959	FACS (CLL staining)	1 in 400
PE-Lambda	Biolegend	407307	FACS (CLL staining)	1 in 400
PE-c-kit	Biolegend	105808	FACS (HSC staining)	1 in 100
PE-Cy7-Sca-1	Biolegend	108114	FACS (HSC staining)	1 in 50
BV421-CD150	Biolegend	115926	FACS (HSC staining)	1 in 25
Alexa 700-CD48	Biolegend	103426	FACS (HSC staining)	1 in 50
BV510-CD45.2	Biolegend	109837	FACS (HSC staining)	1 in 50
PE-Cy7-human CD5	Biolegend	364008	FACS (human CLL staining)	1 in 200
BV421-human CD19	Biolegend	302234	FACS (human CLL staining)	1 in 200
eBioscience™ Fixable Viability Dye eFluor™ 780	Invitrogen™	65-0865-14	FACS (human CLL staining)	1 in1000
APC Annexin V	Biolegend	640920	FACS (human CLL staining)	1 in 200
Anti-Rat-IgG microbeads	Milenty Biotec	130-048-502	MACS	1:30
CD117-microbeads (mouse)	Milenty Biotec	130-091-224	MACS	1:30
H3K27ac	Active Motif	39133	CUT&RUN	0.5μg / reaction
H3K27me3	Cell Signaling Technology	9733S	CUT&RUN	0.5μg / reaction
IgG	Cell Signaling Technology	3900S	CUT&RUN	0.5μg / reaction
CD19 Rat anti-Mouse, Clone: eBio1D3	Fisher Scientific	15286837	MACS	1:200
Parp1 antibody	Cell signalling	9542S	Western blot	1:1500
anti-pan-ADP-ribose	Millipore		Western blot	1:1500
Actin antibody	Santa Cruz	Sc-47778 HRP	Western blot	1:10000
Peroxidase-conjugated AffiniPure™ Goat Anti-Rabbit IgG	Jackson ImmunoResearch	111-035-144	Western blot	1:15000

Supplementary Table S2

Characterization of haematologic malignancies of primary transplanted mice (as shown in Figure 1)

Genotypes	Code	Granulopoiesis	Megakaryocyte	Plt count	RBC count	Hbg count	Splenomegaly	B1α proliferation	Diseases
Asxl1$^{-/-}$Ezh2$^{-/-}$	1078-26	left-shift, Pseudo-Pelger Huet	monolobated and binuclear	normal	normal	normal	yes	yes	MDS/MPN, LPD
Asxl1$^{-/-}$Ezh2$^{-/-}$	1078-27	left-shift	monolobated and multinuclear	high	normal	normal	none	none	MDS/MPN
Asxl1$^{-/-}$Ezh2$^{-/-}$	1092-26	left-shift	monolobated	normal	normal	normal	yes	yes	MDS/MPN, LPD
Asxl1$^{-/-}$Ezh2$^{-/-}$	1092-27	left-shift	monolobated	high	normal	normal	yes	none	MDS/MPN
Asxl1$^{-/-}$Ezh2$^{-/-}$	1093-7	left-shift, Pseudo-Pelger Huet	monolobated and binuclear	high	normal	normal	yes	yes	MDS/MPN, LPD
Asxl1$^{-/-}$Ezh2$^{-/-}$	1092-34	left-shift	Monolobated	normal	normal	normal	yes	yes	MDS/MPN, LPD
Asxl1$^{-/-}$Ezh2$^{-/-}$	1092-35	None	normal shape	high	high	high	none	none	ET-like
Asxl1$^{-/-}$Ezh2$^{-/-}$	1092-36	left-shift, Pseudo-Pelger Huet	bi and multinuclear	normal	normal	normal	yes	yes	MDS/MPN, LPD
Asxl1$^{-/-}$Ezh2$^{-/-}$	8748	left-shift, Pseudo-Pelger Huet	monolobated, and binuclear	normal	normal	normal	yes	none	MDS/MPN
Asxl1$^{-/-}$Ezh2$^{-/-}$	8749	left-shift, Pseudo-Pelger Huet	microform and bionuclear	normal	normal	normal	yes	none	MDS/MPN
Asxl1$^{-/-}$Ezh2$^{-/-}$	8745	left-shift	monolobated	normal	normal	normal	none	none	MDS/MPN
Asxl1$^{-/-}$Ezh2$^{-/-}$	8746	left-shift, Pseudo-Pelger Huet	monolobated	normal	normal	normal	none	none	MDS
Asxl1$^{-/-}$Ezh2$^{-/-}$	8751	left-shift, Pseudo-Pelger Huet	monolobated	normal	normal	normal	yes	none	MDS/MPN
Ezh2$^{-/-}$	1092-17	left-shift, Pseudo-Pelger Huet	monolobated	normal	normal	normal	yes	yes	MDS/MPN, LPD
Ezh2$^{-/-}$	1092-20	left-shift, Pseudo-Pelger Huet	bi and multinuclear	normal	normal	normal	yes	yes	MDS/MPN, LPD
Ezh2$^{-/-}$	1092-24	left-shift	monolobated	normal	normal	normal	yes	yes	MDS/MPN, LPD
Ezh2$^{-/-}$	1078-19	None	normal shape	normal	normal	normal	none	none	None
Ezh2$^{-/-}$	1092-15	None	normal shape	normal	normal	normal	none	none	None
Ezh2$^{-/-}$	1092-19	None	normal shape	normal	high	high	none	none	PV-like
Ezh2$^{-/-}$	8741	left-shift, Pseudo-Pelger Huet	monolobated	normal	high	high	none	none	MDS
Ezh2$^{-/-}$	8740	None	normal shape	normal	normal	normal	none	none	None
Asxl1$^{-/-}$	1078-1	None	normal shape	high	normal	normal	none	none	ET-like
Asxl1$^{-/-}$	1078-2	None	normal shape	normal	normal	normal	none	none	None
Asxl1$^{-/-}$	1078-3	None	normal shape	high	normal	normal	none	none	ET-like
Asxl1$^{-/-}$	1092-4	None	normal shape	normal	high	high	none	none	PV-like
Asxl1$^{-/-}$	1092-11	None	normal shape	normal	high	high	none	none	PV-like
Asxl1$^{-/-}$	1092-12	None	normal shape	normal	normal	normal	none	none	none

Supplementary Table S3

List of primers

Primer	Sequence 5'>3'	Application
Asxl1 fl/wt F (1)	CCAATATGGCCTGGAACTGAC	genotyping
Asxl1 fl/wt R (2)	TAGAGACCAGGTGTGGTGGC	genotyping
Asxl1 del F (3)	AGCACACCAGGCTAAGATGCT	genotyping
Asxl1 del R (4)	ACAGACAAGCAAAGCTGAACAGA	genotyping
Ezh2 fl/wt/del 1	TTATTCATAGAGCCACCTGG	genotyping
Ezh2 fl/wt/del 2	ACGAAACAGCTCCAGATTCAGGG	genotyping
Ezh2 fl/wt/del 3	AGGGCATCAGCCTGGCTGTA	genotyping
VHF1	AGGTCCAGCTGCAGGAGTCTGG	clonality
VHF2	AGGTCCAGCTGCAGGAGTCAGG	clonality
VHF3	AGGTCCAGCTTCAGGAGTCTGG	clonality
VHF4	AGGTCCAGCTTCAGGAGTCAGG	clonality
VHF5	AGGTCCAACTGCAGGAGTCTGG	clonality
VHF6	AGGTCCAACTGCAGGAGTCAGG	clonality
VHF7	AGGTCCAACTTCAGGAGTCTGG	clonality
VHF8	AGGTCCAACTTCAGGAGTCAGG	clonality
VHR2	TGAGGAGACGGTGACCGTGGTCCCTTGGCCCC	clonality
shRunx1	TGCTGTTGACAGTGAGCGCCAGAAGAGTTGTAT CCACAAATAGTGAAGCCACAGATGTATTTGTGG ATACAACTCTTCTGATGCCTACTGCCTCGGA	97mer cloned into SGEP

Supplementary Figure S1 related to Figure 1: (A-D) The analysis of primary transplanted mice at the indicated time points after tamoxifen treatment is shown. (A-B) The dotplots (A) show the percentages of engraftments ($CD45.2^+$), CD45.2 positive myeloid proportion ($Mac1^+$ or/and $Gr1^+$) and lymphoid proportion ($B220^+$, $CD4^+$ or $CD8^+$) in PB, which is further divided into the indicated cell population in (B). (C) The dot plot shows the absolute cell counts in PB. (D) The percentages of G0 ($Ki67^-$), G1 ($Ki67^+DAPI^{low}$) and G2-M-S ($Ki67^+DAPI^{high}$) phases in LSK cells based on Ki67 and DAPI staining are shown. Bar graphs and dot graphs show mean+S.E.M (*P<0.05, **P<0.01 and ***P<0.001, unpaired t-test). (E) Pie charts showing percentages of *Asxl1*$^{-/-}$*, Ezh2*$^{-/-}$ and *Asxl1*$^{-/-}$*Ezh2*$^{-/-}$ mice with specified heterogenous phenotypes. PV-like (polycythaemia vera-like); ET-like (essential thrombocytosis-like); MDS (myelodysplastic syndrome); MDS/MPN (myelodysplastic syndrome/myeloproliferative neoplasm); MDS/MPN, LPD (MDS/MPN and B-cell lymphoproliferative disorder). (F) PCR results confirming the genotypes of Asxl1 and Ezh2 in splenocytes from Asxl1f/fEzh2f/f (WT) and Asxl1 KO (Asxl1$^{-/-}$), Ezh2 KO (Ezh2$^{-/-}$) and double mutant (Asxl1$^{-/-}$Ezh2$^{-/-}$) mice. (G) RNA-seq showing the exon deletions of Asxl1 (exon 3) and Ezh2 (exon 16-19) in BM cells from WT and KO mice.

Supplemental Figure S2 related to Figure 2: (A) Representative flow cytometry profiling of donor cells in bone marrow and spleens of primary transplanted Asxl1$^{-/-}$Ezh2$^{-/-}$ MDS/MPN, LPD mice. (B) ToppGene co-expression atlas showing genes upregulated in bone marrow of the indicated genotypes compared to WT are also expressed in B1a cells. (C) Bar charts showing normalized counts (y-axis) of different IGHV (x-axis) in RNA-seq data of bone marrow cells from normal (WT, Asxl1$^{-/-}$) and MDS/MPN, LPD primary transplanted mice (Ezh2$^{-/-}$ and Asxl1$^{-/-}$Ezh2$^{-/-}$ dKO). (D) RT-PCR of IGHVDJ in splenocytes from normal (WT, Asxl1 KO) and MDS/MPN, LPD primary transplanted mice (Ezh2 KO and Asxl1/Ezh2 dKO), using primer set targeting V and J regions. (E) DNA high sensitivity chip bioanalyzer traces of purified PCR products from (D). (F) 4 colour chromatogram of DNA sequences from purified PCR products from (D). (G) Weights of spleen, liver and BM cellularity (top), WBC, RBC and Hgb (bottom) in WT and Asxl1$^{-/-}$Ezh2$^{-/-}$ secondary CLL mice. (H) Summary of the clonality of secondary and tertiary transplanted CLL mice. (I) Gross pathology of representative control C57BL/6, TCL1 and *Asxl1*$^{-/-}$*Ezh2*$^{-/-}$ tertiary CLL mice. Scale bar, 1cm. Spleens (SP), Liver (LV), Lymph nodes (LN). (J, K, L) The Weight of spleens and

livers (J), the percentage of CD19/CD5 double positive donor cells in spleens (SP), livers (LV), PB and BM (K) and the blood counts (L) in the indicated tertiary mice are shown. (M) Representative images of MG-Giemsa staining of PB from TCL1 and *Asxl1$^{-/-}$Ezh2$^{-/-}$* CLL mice. Typical CLL (1) and large cleaved CLL cells (2) were identified in *Asxl1$^{-/-}$Ezh2$^{-/-}$* CLL mice (Top, red arrows and inset). The percentage of cleaved CLL cells in total *Asxl1$^{-/-}$Ezh2$^{-/-}$* CLL cells (Bottom). Dotplots show mean+SEM (*P<0.05, **P<0.01 and ***P<0.001, unpaired t-test).

Supplemental Figure S3 related to Figure 3: (A) Venn diagram showing the overlaps of DEG (p.adj <0.05) identified in CD19^{+} spleen cells from primary transplanted mice with the indicated genotype, by comparing WT to *Asxl1$^{-/-}$, Ezh2$^{-/-}$* (MDS/MPN, LPD) or *Asxl1$^{-/-}$Ezh2$^{-/-}$* (MDS/MPN, LPD) mice. (B) The bar chart shows example pathways identified in GO and expressed in Asxl1$^{-/-}$Ezh2$^{-/-}$ CD19^{+} cells using DE genes from the Asxl1$^{-/-}$Ezh2$^{-/-}$ dKO vs WT analysis. (C) Example GSEA plots for the indicated pathways in the indicated comparisons are shown. (D) UpSetR plot shows the intersection between the pathways identified in ReactomePA when comparing DEGs highly expressed in CLL comparing 2 models with WT CD19^{+} controls: tertiary TCL1 CLL vs WT or tertiary Asxl1$^{-/-}$Ezh2$^{-/-}$ dKO CLL vs WT. (E) Differential expression analysis (DESeq2) results for the indicated comparisons are shown for certain key DDR genes previously reported being regulated by PcG proteins. (F) Example tracks are shown for the RNA-seq data of the indicated samples (on the left) from BM (top half) or CD19^{+} cells (bottom half) on indicated transposable elements (black box). Blue tracks indicate WT control samples. (G) The genome tracks show examples of a differentially expressed individual ERV (RNA-seq top 2 tracks) and the H3K27me3 and H3K27ac levels in WT and tertiary Asxl1$^{-/-}$Ezh2$^{-/-}$ dKO CLL mice as indicated. The last 2 tracks show control IgG enrichment. (H) The plot shows quantitative H3K27me3 (left) and H3K27ac (right) CUT&RUN data for the indicated TE families for the indicated comparisons: dKO CLL_WT CD19: CD19+ splenocytes isolated from tertiary Asxl1$^{-/-}$Ezh2$^{-/-}$ dKO CLL mice vs CD19+ splenocytes isolated form WT mice. TCL CLL_WT CD19: CD19+ splenocytes isolated from tertiary TCL1 CLL mice vs CD19+ splenocytes isolated form WT mice. TCL CLL_dKO CLL: CD19+ splenocytes isolated from tertiary Asxl1$^{-/-}$Ezh2$^{-/-}$ dKO CLL mice vs CD19+ splenocytes isolated form tertiary TCL CLL mice. (I) Dotplot showing the differential expression (Log2FC) of the indicated TE families in c-kit+ HSPCs (3 months in vivo dataset).

RNAseq data from c-kit+ HSPCs isolated from BM of primary transplanted mice 3 month after tamoxifen treatment, comparing KO vs WT and double KO vs single KO as indicated. (J) ATACseq was performed on control, Asxl1$^{-/-}$, Ezh2$^{-/-}$ and dKO c-kit+ HSPCs harvested from BM of primary transplanted mice 3 months after tamoxifen treatment. The plot shows quantitative ATACseq data (differential expression – Log2FC) for the indicated TE families for the indicated comparisons: Asxl1_WT: Asxl1$^{-/-}$ vs WT, Ezh2_WT: Ezh2$^{-/-}$ vs WT, dKO_WT: dKO vs WT.

Supplemental Figure S4 related to Figure 4: (A) WT and dKO c-kit+ cells were subjected to alkaline and neutral comet assays and the violin plots show the respective tail length (left) and the tail moment (right) results. (B) Dose response curve measuring the relative number of colonies of WT c-kit^{+} cells (n=3) with increasing amounts of Olaparib compared to untreated controls. The EC50 is indicated. (C) Whole comet pictures for the indicated cell types and treatment groups are shown. A cropped section of these pictures is shown in Figure 4E. (D) Comet pictures for the MLL fusion cells with and without PARPi treatment are shown. (E) The dotplot shows the relative number of cells for the indicated genotypes upon the indicated in vitro treatment. PARPi: Olaparib; V: Veliparib (n=2). (F) Schematic diagram of experimental plan to knock down Runx1 in the Asxl1$^{-/-}$Ezh2$^{-/-}$ background. (G) Kaplan-Meier survival curve for the indicated experimental groups (each n=7). (H) The percentage of GFP^{+} donor cells in BM, spleen and liver for each experimental group at the time of sacrifice is shown. (I) TCL1 and Asxl1$^{-/-}$Ezh2$^{-/-}$ CLL cells were treated in vitro with the indicated concentrations of PARPi and stained for AnV/PI. The bar chart depicts the percentage of cells in the indicated stadium. (J) The dotplot shows the relative number of cells for the indicated genotypes upon the indicated in vitro treatment. PARPi: Olaparib 1μM, T: Talazoparib 1μM, V: Veliparib 2μM (n=4). (K) Whole comet pictures for the indicated cell types and treatment groups are shown. A cropped section of these pictures is shown in Figure 4I. (L) Peripheral blood was collected from vehicle or PARPi treated groups right at the end of the treatment course. The dot plots show the Absolute CLL counts, RBC, Hgb and Plt for both groups as indicated. Dotplots show mean+SEM, unpaired t-test.

Supplemental Figure S5 related to Figure 5: (A) Immunofluorescence (left) of WT or dKO c-kit+ cells treated with or without PARPi. Y-H2AX (green), RAD51 (red) and DAPI (blue) were used as indicated. The bar chart on the right summarizes the data from over 100 individual cells and show the percentage of cells with ≥10 y-H2AX foci (top) or the percentage of cells with ≥ 6 RAD51 foci (bottom). (B) Western blot using cell lysates isolated from WT or dKO c-kit+ with anti-Parp1 (top), anti-pan-poly(ADP-ribosyl)ation (middle) and anti-actin (bottom) antibodies as indicated. (C) PCR-based HR efficieny assay (product, manufacturer) with WT and dKO c-kit+ cells with or without PARPi treatment. The HR efficiency percentage is shown in the bar chart for the indicated cell types and conditions. (D) Whole comet pictures for the in vitro deleted Asxl1$^{-/-}$Ezh2$^{-/-}$ c-kit^{+} cells upon the indicated treatment groups are shown. A cropped section of these pictures is shown in Figure 5C. (E) The head length, Tail lengths and Tail moments are shown for the WT c-kit^{+} cells treated with or without PARPi (P), PARPi + didanosine (P+D), PARPi + lamivudine (P+L) as indicated (top). Comet pictures of the cells are shown for each condition (bottom). (F) Tail length and head length measured in alkaline comet assay are shown for dKO c-kit+ cells treated with or without PARPi (P), PARPi + didanosine (P+D), PARPi + lamivudine (P+L) as indicated. (G) The relative number of cells are shown for murine AML1-ETO transformed cells with or without Parpi (P), Parpi + didanosine (P+D) or Parpi + lamivudine (P+L) treatment as indicated for 3 days in vitro. (H) The relative number of colonies (left) are shown for murine AML1-ETO transformed cells with or without Parpi (P), Parpi + didanosine (P+D) or Parpi + lamivudine (P+L) treatment as indicated. Example colony pics are shown (right). (I) After in vitro treatment with or without Parpi, Parpi + didanosine (P+D) or Parpi + lamivudine (P+L) for 24h, murine AML1-ETO cells were subjected to alkaline (top) and neutral (bottom) comet assays and the violin plots show the respective tail length and the tail moment results. (J) The relative number of Asxl1$^{-/-}$Ezh2$^{-/-}$ dKO shRunx1 cells (n=4) are shown following the indicated in vitro treatment (P, PARPi; PD, PARPi + didanosine; PL, PARPi + lamivudine). (K) Whole comet pictures for Asxl1$^{-/-}$Ezh2$^{-/-}$ CLL cells upon the indicated treatment groups are shown. A cropped section of these pictures is shown in Figure 5F. (L) Kaplan Meier survival curves are shown for mice transplanted with dKO CLL cells (same as in Figure 5G) and treated with (n=5) or without RTi (n=10).

Supplemental Figure S6 related to Figure 6: (A) DESeq2 differential expression analysis was carried out for individual copies of TEs between human MDS bone marrow samples with ASXL1/EZH2 (n=3) mutations and human MDS bone marrow samples with other mutations (n=109). Significant copies are defined by adj.p < 0.05 and Log2FC > 1 (UP) or < -1 (DN). (B) Summary of subfamilies represented by more than 3 individual copies being significantly differentially expressed (see Fig S6A). UP: number and identity of subfamilies where > 3 individual copies were UP-regulated in A/Emut MDS. DN: number and identity of subfamilies where >3 individual copies were DN-regulated in A/Emut MDS. (C) Example genome tracks showing RNA expression of A/Emut MDS (n=3) and other MDS (n=13, randomly selected) as indicated. (D) Wald-statistics are shown for LINE1 (L1) transposable elements for different comparisons in the MDS dataset (n=112, Figure 6A). Top, data shown for comparison of samples with ASXL1 mutations (n=18, no additional EZH2 mutation) and human MDS bone marrow samples with other mutations; bottom, data shown for comparison of samples with EZH2 mutations (n=3, no additional ASXL1 mutations) and human MDS bone marrow samples with other mutations. (E) Whole comet pictures for the indicated human patient samples with or without the indicated treatments are shown. A cropped section of these pictures is shown in Figure 6E. (F) The dotplot shows the relative number of cells after the indicated treatment for MDS A/Emut (human MDS sample with ASXL1 and EZH2 mutation) (n=4) and MLLr (human MLL-rearranged AML) (n=2). PARPi: Olaparib 1uM; T: talazoparib 1uM; V: veliparib 1uM. (G) The bar chart shows the percentage of MDS A/Emut cells with ≥10 yH2AX foci upon the indicated treatments. (H) Example merged (y-H2AX: green, DAPI: blue) pictures for MDS A/Emut cells for the indicated treatments are shown. (I) Comet pictures for MDS A/Emut cells for the indicated treatments are shown. (J) Representative pictures of y-H2AX (green) and DAPI (blue) stained human ASXL mutated CLL (ASXL1) or human TP53 mutated CLL (TP53) with or without PARPi treatment as indicated. (K) Whole comet pictures for the ASXL1mut human CLL with or without the indicated treatments are shown. A cropped section of these pictures is shown in Figure 6J. Dotplots show mean+SEM, unpaired t-test.

Online Methods

Cell lines

MS-5 cells (DMSZ) were grown in IMDM containing 15% FBS and 1% Pen/strep. CD40L expressing fibroblasts [1] were cultured in RPMI containing 10% FBS and 1% Pen/strep. Cells were expanded and irradiated with 75 grays. Cells were immediately frozen and stored in LN2. All cell lines were maintained in a humidified 5% CO2 incubator at 37°C.

Mice

Compound *Asx1*$^{fl/fl/}$*Ezh2*$^{fl/fl}$ Rosa26-CreER mice were generated by back crossing *Ezh2*^{tm1Tara} (MGI2661097) [2] Gt(ROSA)26Sor$^{tm1(cre/ESR1)Tyj}$ (MGI:3699244) with *Asxl1*$^{tm1c(EUCOMM)Wtsi}$ [3] in C57BL/6J (CD45.2) background, in which critical exons (exon 16 to 19 in *Ezh2* and exon 3 in *Asxl1*) are flanked with loxP sites. C57BL/6 mice congenic for the Ly5 locus (CD45.1) B6.SJL-Ptprca Pepcb/BoyJ were used as recipient mice. Asxl1$^{tm1a(EUCOMM)Wtsi}$ mice (MGI44878) were initially crossed to a germline Flpase-deleter in order to remove marker cassette to generate Asxl1$^{tm1c(EUCOMM)Wtsi}$ as previously described [3 4-6], which are denoted as floxed Asxl1 mice in the manuscript. Genotyping PCRs were performed on genomic DNA isolated from cells or ear snips as previously described (Table S3).

Bone marrow (BM) transplantation

$2x10^6$ BM cells collected from $CD45.2^+$ donor mutant mice were intravenously (i.v.) transplanted into $CD45.1^+$ recipient mice with lethal irradiation (13.5 Gy) together with 0.2 million rescue BM cells from recipient mice via tail veins in primary experiments, and subsequently, transplanted mice were injected with 120 µl tamoxifen (10 mg/ml) intraperitoneally (i.p.) one month after transplantation for 5 consecutive days to activate Rosa26-CreER for knocking out *Asxl1* and/or *Ezh2*. For the following serial transfers, instead of lethal irradiation, 1×10^5 BM cells or splenocytes from leukaemic mice were transplanted into sublethally (11Gy) irradiated $CD45.1^+$ recipient mice. The chimeric status of donor-derived haematopoietic cells was monitored through flow cytometric analysis of CD45.1 and CD45.2, and the ratios of donor cells were evaluated by means of dividing $CD45.2^+$ cells into the sum of total $CD45^+$ cells. Log-rank (Mantel-Cox) test was performed to determine statistical significance survival

differences between 2 groups. The researchers were not blinded to the experimental groups.

Runx1 knock down in Asxl1$^{-/-}$Ezh2$^{-/-}$ c-kit^{+} cells

Asx1$^{fl/f/}$*Ezh2*$^{fl/fl}$ Rosa26-CreER mice were treated in vivo for 4 days with 120 µl tamoxifen (10 mg/ml) intraperitoneally (i.p.) and c-kit^{+} cells were isolated (Miltenyi Biotec) 3 days after the last injection. Cells were cultured overnight (o/n) in Stemspan (Stemcell Technologies) supplemented with 20ng/ml SCF, 10ng/ml IL3 and 10ng/ml IL6. The following day cells were transduced with lentivirus expressing shRNA targeting Runx1 (Table S3) (SGEP-shRunx1) or empty vector (SGEP). 24h later GFP^{+} cells were sorted and 20k GFP^{+} cells of each group were transplanted into sublethally irradiated mice. GFP was used as a marker to distinguish donor from host cells. Log-rank (Mantel-Cox) test was performed to determine statistical significance survival differences between 2 groups.

Drugs and cytokines

Didanosine, Lamivudine, Veliparib were purchased from ApexBio, Talazoparib from Selleckchem, HBC from Sigma and Olaparib from LC laboratories or ThermoFisher Scientific.

Human SCF, TPO, FLT3L, IL3, IL6, IL21 and mouse IL4, SCF, IL3, IL6, GM-CSF were purchased from Miltenyi Biotec.

In vivo treatment

1×10^5 BM cells isolated from an *Asxl1*/*Ezh2* dKO CLL mouse in secondary experiments were i.v. injected into each sublethally irradiated (11 Gy) CD45.1^{+}SJL recipient mouse via tail veins. Vehicles (10% HBC), 50 mg/kg olaparib were given to mice intraperitoneally one week after transplantation at 5 days per week for 4 weeks. RT inhibitor (RTi) Lamivudine was given in drinking water at 2mg/ml for 4.5 weeks starting 5 days before first olaparib injection.

Flow cytometry and antibodies

All antibodies are listed in Table S1. BM cells, splenocytes and PB cells suspended in PBS with 2% FBS (SM buffer) after 10 mins red blood cell lysis were stained with a

general staining cocktail comprised of antibodies binding CD45.1, CD45.2, Mac1 (CD11b), Gr1, B220, CD4, CD8 and c-kit to divide myeloid populations (Mac1$^+$ or/and Gr1$^+$), total lymphoid populations (Mac1$^-$Gr1$^-$), normal B cells (B220) and T cells (CD4/8) and the CLL staining cocktail comprised of antibodies targeting CD45.2, CD19, CD5, B220, CD43 and IgM at 1 in 400 dilution for 30 mins. Cell populations were analysed by fluorescence-activated cell sorting (FACS)-LSR II (BD, USA). All antibodies for FACS were purchased from BioLegend. HSPC staining was performed as we previously described [6,7]. Briefly, 4×10^6 BM cells after lysis of red blood cells were resuspended in 200 µl SM buffer and subjected to incubation of unconjugated rat IgG (7 Ab: Ter119, Mac1, Gr1, CD3ε, CD4, CD8, B220) in 40X dilution for 30 mins at 4°C, followed by incubation of Lin Ab Texas-Red (45X dilution) for another 30 mins at 4°C in the dark after wash and centrifuge at 1500 rpm for 8 mins. Furthermore, BM cells were subjected into HSPC Ab staining for 30 mins at 4°C in the dark with indicated antibody dilution and specific cell types of HSPCs were gating based on the cell markers[8,9]. To determine the number of B-cells in human CLL samples, cells were stained with anti-human CD5 and CD19 antibodies, fixable viability dyes eFluor™ 780 and Annexin V in Annexin V binding buffer for 30 minutes at room temperature in the dark. Cells were washed with Annexin V binding buffer and analysed using a LSR II (BD, USA). The percentage of live CD19+CD5+ cells was then multiplied with the number of cells determined by trypan blue staining. Unpaired, two-sided Student's t-test were performed to determine statistical significance between two groups.

May-Grunwald (MG)/ Giemsa staining and identification of dysplastic cells

Cytospin of 1×10^5 BM cells and PB smears were stained with May-Grunwald (Sigma Aldrich) for 5 mins, followed by Giemsa staining at 10× dilution for 15 mins, and staining slides were washed with water and dried on air at room temperature. Finally, staining samples were fixed and covered with mounting medium for microscopic observation. Cells amongst granulocytic maturation and myeloid dysplastic cells were identified by cell morphology according to a previous cytologic studies[10], and left-shift of granulopoiesis was determining based on proportions of immature granulocytes.

Amplification and analysis of IGHV-IGHD-IGHJ gene rearrangements

RNA was extracted from splenocytes in primary experiments using RNEasy mini kit (QIAGEN), and 100ng RNA was subjected to reverse transcription under 20 µl reaction

containing 1X First Strand Buffer, 200 units of Superscript III reverse transcriptase (Thermo Fisher Scientific), 0.5 mM dNTP, 100 pmol random hexamers (N6, NEB), 1 unit of RNAse inhibitors and 5 mM DTT at 50°C for 1 hr. 2 µl cDNA was amplified using a published primer set targeting mouse *IGHV* (forward) and *IGHJ* (reverse*)* (12 pmol VHR2 and 24 pmol VHF1-8, Table S3)[11,12] with 0.2 mM dNTP, 1.5 mM MgSO4, 3 units of Dream Taq DNA pol in a 50 µl volume for 35 cycles, each of which consisting of 30s denaturation at 94 °C, 30s annealing at 50°C and 1 min elongation at 72°C, followed by 10 min extension at 72°C. 10 ng/µl PCR products extracted from 2 % agarose gel using QIAquick Gel Extraction kit (QIAGEN) were sent for Sanger sequencing (Source BioScience), and IGHVDJ *and* HCDR3 were identified using IMGT V-QUEST tool (https://www.imgt.org/IMGT_vquest/input).

RNA-seq library preparation

RNA was isolated and either subjected to in house cDNA library prep using the TruSeq Stranded Total RNA LT Kit with RiboZero Gold according to manufacturer's protocol (Illumina, Inc) or send to Novogene for library preparation. From the primary transplanted mice, RNA was extracted from BM cells with over 90% engraftment in the wild type and mutant mice using RNEasy mini kits (QIAGEN). Splenic $CD19^+/CD45.2^+$ donor B cells from primary or tertiary mice were sorted by Aria FACS sorter (BD, USA), while normal $CD19^+$ B cells from wild type mice were purified by MACS separation using anti-Rat IgG microbeads (Miltenyi Biotec Cat. No 130-048-501, Germany) and RNA was extracted using Monarch Total RNA Miniprep kits (NEB #T2010, USA). 10 µl RNA from each spleen sample was subjected to preparation of cDNA library using the NEBNext Poly(A) Magnetic Isolation Module according to manufacturer's protocol (NEB #E7490, USA). Bone marrow $CD45.2^+$/ c-kit$^+$ Asxl1$^{-/-}$ Ezh2$^{-/-}$donor cells were FACS sorted from short-term (3 months) in vivo deleted mice and RNA was isolated using Monarch Total RNA Miniprep kits and send to Novogene for library preparation. RNA was isolated from very short term in vivo deleted c-kit$^+$ Asxl1$^{-/-}$Ezh2$^{-/-}$ cells using Monarch Total RNA Miniprep kits and send to Novogene for library preparation.

Cleavage Under Targets & Release Using Nuclease (CUT&RUN) sequencing

CUT&RUN was performed according to Epicypher CUT&RUN Protocol V2.0 as previously described [6]. Briefly, 0.5 million Asxl1$^{-/-}$Ezh2$^{-/-}$ dKO tertiary CLL or $CD19^+$

WT splenocytes were rinsed with wash buffer (20 mM HEPES, pH 7.5, 150 mM NaCl, 0.5 mM Spermidine, and 1× Complete Protease Inhibitor Cocktail) and incubated with the activated Concanavalin A (ConA) beads (Bangs Laboratories BP531) at RT for 10 min. 0.5 µl antibody (H3K27me3 Cell Signaling Technology C36B11; H3K27ac Active Motif 39133 or IgG Millipore 12-370) was added and incubated at 4 °C overnight. After permeabilization with digitonin buffer (wash buffer plus 0.01% digitonin), 50 ng pAG-MNase (kindly provided by Dr Paul Lavender, King's College London) was added and bound to antibody labelled chromatin. MNase was subsequently activated by addition of CaCl2 to cleave and release antibody-bound chromatin. DNA purification was performed using Monarch PCR cleanup kit (NEB, Monarch PCR & DNA Cleanup Kit, T1030), DNA libraries were prepared using the NEB Ultra II DNA Library Prep Kit (NEB#E7103). Each condition and cell type were assayed twice and distinct samples were used.

In vitro culture mouse CLL cells

Asxl1$^{-/-}$Ezh2$^{-/-}$ dKO CLL or TCL1 CLL cells were cultured in IMDM, 10%FBS, pen/Strep, non-essential amino acids (NEAA), 0.3mM 2-mercaptoethanol, and 10 ng/ml IL4 and 25 ng/ml IL21 in the presence or absence of 1mM olaparib, 25mM didanosine, 25mM lamivudine, 1mM talazoparib, 1mM veliparib as indicated for 48-72h. Distinct samples were measured. Unpaired, two-sided Student's t-test were performed to determine statistical significance between two groups.

Isolation and in vitro culture mouse c-kit^{+} cells

c-kit^{+} cells were isolated from bone marrow of WT or Asxl1$^{fl/fl}$Ezh2$^{fl/fl}$ RosaCreER mice using CD117 microbeads (Miltenyi Biotec) and cultured in RPMI, 10%FBS, Pen/Strep, 20ng/ml SCF, 10ng/ml IL3 and 10ng/ml IL6. In vitro deletion of floxed alleles was achieved by adding 25nM 4-hydroxytamoxifen (4-OHT) for 3 days. Cells were then washed with PBS and cultured for 48-72h (without 4-OHT) with or without 1mM Olaparib, 25mM didanosine, 25mM lamivudine, 1mM talazoparib, 1uM veliparib as indicated. Alternatively, after the 4-OHT incubation, 5000-10000 cells were plated in methylcellulose (M3434, Stemcell) supplemented with 20 ng/ml SCF and 10 ng/ml of each IL3, IL6 and GM-CSF with or without 1mM Olaparib, 25mM didanosine, 25mM lamivudine, 1mM talazoparib, 1mM veliparib as indicated. Colonies were stained after 7 days with INT and scored. Distinct samples were measured. Unpaired, two-sided

Student's t-test were performed to determine statistical significance between two groups.

Culture of AML1-ETO cells

Primary murine AML1-ETO transformed cells were generated as described before [13]. For cell growth experiments, AML1-ETO cells were cultured for 72h (24h for comet assay) with or without 1mM Olaparib, 25mM didanosine, 25mM lamivudine as indicated. Alternatively, 5000 cells were plated in methylcellulose (M3434, Stemcell) supplemented with 20 ng/ml SCF and 10 ng/ml of each IL3, IL6 and GM-CSF with or without 1mM Olaparib, 25mM didanosine, 25mM lamivudine as indicated. Colonies were stained after 7 days with INT and scored. Distinct samples were measured. Unpaired, two-sided Student's t-test were performed to determine statistical significance between two groups.

Human cell culture

Human MDS and AML cells were plated on MS5 stroma cells and cultured in IMDM, 15%FBS, Pen/Strep, 10ng/ml of each SCF, TPO, FLT3L, IL3 and IL6 with or without 1mM Olaparib, 25mM didanosine, 1mM talazoparib, 1mM veliparib as indicated for 6 days. One ASXL1/EZH2mut MDS sample and one MLLr AML sample were repeatedly measured.

Human CLL cells were transferred onto irradiated CD40L-expressing fibroblasts [1] (which were thawed from LN2 and plated the day before) and cultured in IMDM, 10%FBS, pen/Strep, NEAA, 0.3mM 2-mercaptoethanol, 10 ng/ml IL4 and 25 ng/ml IL21 for 5 days. On day 5, 1mM olaparib and 25mM didanosine was added to the respective treatment wells while controls did not receive any drugs and cells were harvested for analysis after 2-6 days of drug treatment. Three ASXL1mut CLL and one P53mut CLL sample were used.

Unpaired, two-sided Student's t-test were performed to determine statistical significance between two groups.

Homologous recombination assay by qPCR

Homologous Recombination Assay Kit (Norgen Biotek; Cat No. 35600) was used to quantify the efficiency of homologous recombination (HR) by quantitative PCR

(qPCR), according to the manufacturer protocol. Briefly, to transfect the plasmids for HR assay by electroporation (Invitrogen Neon™ Transfection System; Cat No. MPK5000), 500K to 1million cells were harvested, washed once with PBS, and then resuspend into 100ul Buffer T with 10ul each D1 and D2 plasmids. Cells were incubated at room temperature for 20 minutes, and then electroporated with programme of 1550V, 10ms interval, for 3 pulses. Then cells were transferred to 2mL of culturing medium and placed in CO2 incubator for 1 hour to recover. Cells were then split into 2 wells and treated with/ without 1uM Olaparib for 24 hours. After drug treatment, plasmid DNA were then harvested using genomic DNA extraction kit (New England Biolab; Cat No. T3010). Extracted DNA was then subjected to qPCR using SybrBreen (Applied Biosystems™; Fast SYBR™ Green Master Mix; Cat No. 4385612) with StepOnePlus Real-Time PCR Systems using the PCR programme according to HR assay kit.

Western Blot

Total cell lysate of WT and dKO c-kit+ cells was separated on an 8% gel by SDS-PAGE and transferred onto a PVDF membrane. After blocking, membrane was incubated overnight with primary antibodies detecting the proteins of interest. After secondary antibody incubation and wash steps, ECL was added, and proteins were visualised using chemiluminescence. Antibodies used for Western blotting are shown in Table 1.

Comet assay

The Comet Assay Kit (Cat: 4250-050-K) from R&D Systems, Minneapolis, MN, USA, was used according to the manufacturer instructions. Briefly, 20k cells were washed, resuspended in 25ul PBS and combined with LM (low melting) Comet Agarose at a volumetric ratio of 1:10 (cell pellet:agarose), and 50 µL of the mixture was dispensed into each well of the Comet slide. Following complete drying at 4°C in the dark for 30 minutes, the slides were incubated with precooled Lysis solution overnight, maintaining darkness throughout the process. For neutral comet assay, slides were then immersed in neutral electrophoresis buffer (50 mM Tris Base, 300 mM sodium acetate trihydrate) for 30 minutes and electrophoresis was performed in the same buffer at 18 V for 40 minutes. For alkaline comet assay, slides were instead immersed into alkaline unwinding solution for 1 hour at 4°C and electrophoresis was performed

in alkaline electrophoresis buffer at 18 V for 40 minutes. After electrophoresis, slides were incubated with 70% EtOH for 30 minutes (neutral comet) or 5 minutes (alkaline comet) and subsequently dried at 37C. Then the slides were stained with SYBR Gold dye for 45 minutes at room temperature, washed with sterile distilled water, and examined under an EQ Leica DM4000 fluorescence microscope (Leica Microsystems Inc). When assessing the degree of DNA damage, at least 15 randomly selected microscopic fields were imaged per slide covering a minimum of 500 cells in total. Image analysis was conducted utilizing the OpenComet plug-in of ImageJ software (version 1.45s, National Institute of Health, USA, RRID: SCR 003070). All quantitative data underwent analysis using GraphPad Prism version 10.1 software (RRID: SCR_002798). To assess distribution normality, the D'Agostino-Pearson test was employed. For data sets that showing a normal distribution, One-Way ANOVA was employed, whereas for datasets exhibiting non-normal distribution, the Kruskal-Wallis test was utilized for statistical analysis. Tukey's posthoc test was subsequently applied to determine significant differences among groups. Statistical significance was defined as *p<0.05, **p<0.01, ***p<0.001, ****p<0.0001. At least two experiments were performed and one representative result is shown in the figure panels.

y-H2AX / Rad51 staining

Briefly, up to 100K cells were cytospun onto a slide. Cells were fixed using 4% paraformaldehyde at room temperature for 10 mins, permeabilized using 0.1% Triton X-100 for 5 mins, and then blocked with 1% bovine serum albumin for 30 mins. Cells were then stained with gH2AX antibody at the dilution of 1:200-1:500 and Rad51 antibody at the 1:333 dilution overnight at 4°C, followed by secondary Alexa-488 conjugated goat-anti-mouse antibody at the dilution of 1:400 and secondary Cy3 conjugated goat-anti-rabbit antibody at the dilution of 1:333 together with 1ug/mL DAPI for 1-3 hours at room temperature. Cells were then mounted and examined under an EQ Leica DM4000 fluorescence microscope (Leica Microsystems Inc). When assessing the degree of DNA damage, 5-10 randomly selected microscopic fields were imaged per slide covering a minimum of 200 cells. All cells within these fields were counted, and those exhibiting 10 or more foci were classified as damaged. The percentage of damaged cells within each microscopic field was subsequently calculated. Statistical analysis was performed on the percentage of damaged cells across the sampled fields. The D'Agostino-Pearson test was utilized to evaluate the

normality of the data distribution. For data that showed a normal distribution, One-Way ANOVA was employed for comparative analysis, followed by Tukey's post hoc test to identify specific intergroup differences. Statistical significance was defined as *p<0.05, **p<0.01, ***p<0.001, ****p<0.0001. At least two experiments were performed, and one representative result is shown in the figure panels.

Bioinformatic analysis

Once sequenced, NGS PE150 RNA-seq reads were aligned using the STAR aligner (RRID: SCR_004463) [14] to mm10 (mouse samples) or hg38 (human samples) reference genomes. BAM files were then converted to BigWig files using Bedtools [15] and bedgraphToBigWig (from UCSC Utilities) for visualisation as custom tracks in UCSC genome viewer or in IGV (integrated genome viewer). TE locations were identified using RepeatMasker [16] and used by the featureCount function (RRID: SCR_012919) of Rsubread [17] to give read counts at all known TEs. Genes were also counted in the same way using the mm10 or hg38 references. Read counts for individual TEs were pooled into both families and subfamilies for further analysis. For TE family and subfamily analysis, we included multi-mapped reads aggregated from all copies of each TE subfamily as previously described [18]. The 4 human CLL samples sequenced by us were added to a larger human CLL dataset (phs000922) which was downloaded from dbGaP. The human MDS BMMNC dataset was obtained from Seishi Ogawa [19]. Differential Expression was carried out on the combined TE and Gene read counts using DESeq2 [20] weighted using Independent Hypothesis Weighting (IHW) [21]. Significantly expressed TEs, were identified based on an adjusted p-value <= 0.01, log2FoldChange >= ±1.5 and a maximum RPM >= 1000 (mouse) or adjusted p-value <0.05, log2FoldChange >±1 (human). Ranked gene lists based on the log2fold change computed in the DESeq2 analysis were used for gene set enrichment analysis (GSEA,) [22] using a custom gene set list comprising of the misigdb (RRID: SCR_022870) [22-24] Hallmark and Reactome subset of Canonical pathways of the C2 gene set collection. Gene set variation analysis (GSVA, RRID: SCR_021058) [25] was performed on TPM normalized read counts using the same custom gene set list described above. Mean enrichment scores for the indicated groups were plotted in the heatmap for the reported gene sets. Plots were created using ggplot2 [26]. Cut&Run analysis was conducted using the CUT&RUNTools pipeline [27] with default parameters. Read counts for individual TEs were pooled into subfamilies as described above and

H3K27me3 and H3K27ac binding fold changes were calculated from E.coli spike-in reads normalized read counts. BigWig files were normalized to E.coli spike in reads and aggregate tracks were shown. ATACseq was performed and analysed as previously described[6]. Read counts were aggregated to generate a gene count and a TE count table as described above. Differential Expression was carried out as described above.

Biological materials availability

All stable and unique reagents generated in this study are available from the lead contact upon reasonable request.

References

1. Hamilton E, Pearce L, Morgan L, et al. Mimicking the tumour microenvironment: three different co-culture systems induce a similar phenotype but distinct proliferative signals in primary chronic lymphocytic leukaemia cells. *Br J Haematol*. Sep 2012;158(5):589-99. doi:10.1111/j.1365-2141.2012.09191.x
2. Su IH, Basavaraj A, Krutchinsky AN, et al. Ezh2 controls B cell development through histone H3 methylation and Igh rearrangement. *Nat Immunol*. Feb 2003;4(2):124-31. doi:10.1038/ni876

ni876 [pii]
3. Rohatgi N, Zou W, Collins PL, et al. ASXL1 impairs osteoclast formation by epigenetic regulation of NFATc1. *Blood Adv*. Oct 9 2018;2(19):2467-2477. doi:10.1182/bloodadvances.2018018309
4. Skarnes WC, Rosen B, West AP, et al. A conditional knockout resource for the genome-wide study of mouse gene function. *Nature*. Jun 15 2011;474(7351):337-42. doi:10.1038/nature10163
5. White JK, Gerdin AK, Karp NA, et al. Genome-wide generation and systematic phenotyping of knockout mice reveals new roles for many genes. *Cell*. Jul 18 2013;154(2):452-64. doi:10.1016/j.cell.2013.06.022
6. Lynch J, Troadec E, Fung TK, et al. Hematopoietic stem cell quiescence and DNA replication dynamics maintained by the resilient beta-catenin/Hoxa9/Prmt1 axis. *Blood*. Apr 18 2024;143(16):1586-1598. doi:10.1182/blood.2023022082
7. Smith LL, Yeung J, Zeisig BB, et al. Functional crosstalk between Bmi1 and MLL/Hoxa9 axis in establishment of normal hematopoietic and leukemic stem cells. *Cell Stem Cell*. Jun 3 2011;8(6):649-62. doi:10.1016/j.stem.2011.05.004

S1934-5909(11)00224-4 [pii]
8. Doulatov S, Notta F, Laurenti E, Dick JE. Hematopoiesis: a human perspective. *Cell Stem Cell*. Feb 3 2012;10(2):120-36. doi:10.1016/j.stem.2012.01.006

9. Pietras EM, Reynaud D, Kang YA, et al. Functionally Distinct Subsets of Lineage-Biased Multipotent Progenitors Control Blood Production in Normal and Regenerative Conditions. *Cell stem cell*. Jul 2 2015;17(1):35-46. doi:10.1016/j.stem.2015.05.003
10. Zhou T, Kinney MC, Scott LM, Zinkel SS, Rebel VI. Revisiting the case for genetically engineered mouse models in human myelodysplastic syndrome research. *Blood*. Aug 27 2015;126(9):1057-68. doi:10.1182/blood-2015-01-624239
11. White HN. Restriction-PCR fingerprinting of the immunoglobulin VH repertoire: direct detection of an immune response and global analysis of B cell clonality. *European journal of immunology*. Oct 1998;28(10):3268-79. doi:10.1002/(SICI)1521-4141(199810)28:10<3268::AID-IMMU3268>3.0.CO;2-F
12. Yan XJ, Albesiano E, Zanesi N, et al. B cell receptors in TCL1 transgenic mice resemble those of aggressive, treatment-resistant human chronic lymphocytic leukemia. *Proceedings of the National Academy of Sciences of the United States of America*. Aug 1 2006;103(31):11713-8. doi:10.1073/pnas.0604564103
13. Esposito MT, Zhao L, Fung TK, et al. Synthetic lethal targeting of oncogenic transcription factors in acute leukemia by PARP inhibitors. Research Support, Non-U.S. Gov't. *Nat Med*. Dec 2015;21(12):1481-90. doi:10.1038/nm.3993
14. Dobin A, Davis CA, Schlesinger F, et al. STAR: ultrafast universal RNA-seq aligner. *Bioinformatics*. Jan 1 2013;29(1):15-21. doi:10.1093/bioinformatics/bts635
15. Quinlan AR, Hall IM. BEDTools: a flexible suite of utilities for comparing genomic features. *Bioinformatics (Oxford, England)*. 2010;26(6):841-842. doi:10.1093/bioinformatics/btq033
16. Smit A, Hubley, R & Green, P. . RepeatMasker Open-4.0. *2013-2015* <http://wwwrepeatmaskerorg>.
17. Liao Y, Smyth GK, Shi W. The R package Rsubread is easier, faster, cheaper and better for alignment and quantification of RNA sequencing reads. *Nucleic Acids Res*. May 7 2019;47(8):e47. doi:10.1093/nar/gkz114
18. Karimi MM, Goyal P, Maksakova IA, et al. DNA methylation and SETDB1/H3K9me3 regulate predominantly distinct sets of genes, retroelements, and chimeric transcripts in mESCs. *Cell Stem Cell*. Jun 3 2011;8(6):676-87. doi:10.1016/j.stem.2011.04.004
19. Shiozawa Y, Malcovati L, Galli A, et al. Gene expression and risk of leukemic transformation in myelodysplasia. *Blood*. Dec 14 2017;130(24):2642-2653. doi:10.1182/blood-2017-05-783050
20. Love MI, Huber W, Anders S. Moderated estimation of fold change and dispersion for RNA-seq data with DESeq2. *Genome biology*. 2014;15(12):550. doi:10.1186/s13059-014-0550-8
21. Ignatiadis N, Klaus B, Zaugg JB, Huber W. Data-driven hypothesis weighting increases detection power in genome-scale multiple testing. *Nat Methods*. Jul 2016;13(7):577-80. doi:10.1038/nmeth.3885
22. Subramanian A, Tamayo P, Mootha VK, et al. Gene set enrichment analysis: a knowledge-based approach for interpreting genome-wide expression profiles. *Proceedings of the National Academy of Sciences of the United States of America*. 2005;102(43):15545-15550. doi:10.1073/pnas.0506580102
23. Liberzon A, Subramanian A, Pinchback R, Thorvaldsdottir H, Tamayo P, Mesirov JP. Molecular signatures database (MSigDB) 3.0. *Bioinformatics*. Jun 15 2011;27(12):1739-40. doi:10.1093/bioinformatics/btr260
24. Castanza AS, Recla JM, Eby D, Thorvaldsdottir H, Bult CJ, Mesirov JP. Extending support for mouse data in the Molecular Signatures Database (MSigDB). *Nat Methods*. Nov 2023;20(11):1619-1620. doi:10.1038/s41592-023-02014-7
25. Hanzelmann S, Castelo R, Guinney J. GSVA: gene set variation analysis for microarray and RNA-seq data. *BMC Bioinformatics*. Jan 16 2013;14:7. doi:10.1186/1471-2105-14-7

26. Wickham H. ggplot2: Elegant Graphics for Data Analysis. *Springer-Verlag New York*. 2016;
27. Zhu Q, Liu N, Orkin SH, Yuan GC. CUT&RUNTools: a flexible pipeline for CUT&RUN processing and footprint analysis. *Genome Biol*. Sep 9 2019;20(1):192. doi:10.1186/s13059-019-1802-4
